# Differentiation, ageing and leukaemia alter the metabolic profile of human bone marrow haematopoietic stem and progenitor cells

**DOI:** 10.1038/s41556-025-01709-7

**Published:** 2025-07-15

**Authors:** Maria-Eleni Lalioti, Mari Carmen Romero-Mulero, Noémie Karabacz, Julian Mess, Helen Demollin, Jasmin Rettkowski, Konrad Schuldes, Michael Mitterer, Carolin Wadle, Khalid Shoumariyeh, Mirijam Egg, Carlos Alfonso-Gonzalez, Karin Jäcklein, Katharina Schönberger, Nikolaos Karantzelis, Gregor Reisig, Philipp Aktories, Isabella M. Mayer, Ioanna Tsoukala, Alexander Schäffer, Irene Tirado-Gonzalez, Aurélien Dugourd, Lukas M. Braun, Beatriz Silva-Rego, Michael-Jason Jones, Katrin Kierdorf, Julio Saez-Rodriguez, Kilian Reising, Sebastian Gottfried Walter, Hind Medyouf, Valérie Hilgers, Gabriel Ghiaur, Robert Zeiser, Darja Karpova, Simon Renders, Sascha Gravius, Joerg Buescher, Nina Cabezas-Wallscheid

**Affiliations:** 1 Max Planck Institute of Immunobiology and Epigenetics, Freiburg, Germany; 2 Laboratory of Stem Cell Biology and Ageing, Department of Health Sciences and Technology, Swiss Federal Institute of Technology (ETH Zürich), Zurich, Switzerland; 3 University of Freiburg, Faculty of Biology, Freiburg, Germany; 4 International Max Planck Research School of Immunobiology, Epigenetics and Metabolism, Freiburg, Germany; 5 Centre for Integrative Biological Signalling Studies, Freiburg, Germany; 6 Department of Medicine I – Medical Center, Faculty of Medicine, University of Freiburg, Freiburg, Germany; 7 German Cancer Consortium (DKTK), Partner Site Freiburg, a Partnership Between DKFZ and Medical Center, University of Freiburg, Freiburg, Germany; 8 Laboratory of Exercise and Health, Department Health Sciences and Technology, Swiss Federal Institute of Technology (ETH Zürich), Zurich, Switzerland; 9 Department of Orthopaedic and Trauma Surgery, University Medical Centre Mannheim, Medical Faculty Mannheim, University of Heidelberg, Mannheim, Germany; 10 Institute of Neuropathology, Faculty of Medicine, University of Freiburg, Freiburg, Germany; 11 Georg-Speyer-Haus, Institute for Tumor Biology and Experimental Therapy, Frankfurt am Main, Germany; 12 Faculty of Medicine, and Heidelberg University Hospital, Institute for Computational Biomedicine, Heidelberg University, Heidelberg, Germany; 13 Department of Orthopedic Surgery and Traumatology, Medical Center, Faculty of Medicine, University of Freiburg, Freiburg im Breisgau, Germany; 14 Department of Orthopedics, Traumatology and Reconstructive Surgery, University Hospital Cologne, Cologne, Germany; 15 Frankfurt Cancer Institute, Frankfurt am Main, Germany; 16 Department of Hematology Oncology, University Hospital Aachen, Aachen, Germany; 17 Division of Hematological Malignancies, Department of Oncology, Sidney Kimmel Comprehensive Cancer Center, Johns Hopkins University Hospital, Baltimore, MD USA; 18 Institute for Transfusion Medicine and Immunohematology, Goethe University Hospital Medical School, German Red Cross Blood Donor Service, Frankfurt am Main, Germany; 19 Department of Internal Medicine V, Hematology, Oncology and Rheumatology, Heidelberg University Hospital, Heidelberg, Germany

**Keywords:** Haematopoietic stem cells, Quiescence

## Abstract

Metabolic cues are crucial for regulating haematopoietic stem and progenitor cells (HSPCs). However, the metabolic profile of human HSPCs remains poorly understood due to the limited number of cells and the scarcity of bone marrow samples. Here we present the integrated metabolome, lipidome and transcriptome of human adult HSPCs (lineage^−^, CD34^+^, CD38^−^) upon differentiation, ageing and acute myeloid leukaemia. The combination of low-input targeted metabolomics with our newly optimized low-input untargeted lipidomics workflow allows us to detect up to 193 metabolites and lipids from a starting material of 3,000 and 5,000 HSPCs, respectively. Among other findings, we observe elevated levels of the essential nutrient choline in HSPCs compared with downstream progenitors, which decline upon ageing and further decrease in acute myeloid leukaemia. Functionally, we show that choline supplementation fuels lipid production in HSPCs and enhances stemness. Overall, our study provides a comprehensive resource identifying metabolic changes that can be utilized to promote and enhance human stem cell function.

## Abstract (web summary)

Lalioti, Romero-Mulero et al. combine metabolomics, lipidomics and transcriptomics of haematopoietic stem and progenitor cells during differentiation, ageing and leukaemia, finding a role for choline and showing that supplementation enhances stemness.

## Main

Haematopoietic stem cells (HSCs) represent a rare cell population that resides in the bone marrow (BM) at the top of the haematopoietic hierarchy and harbours the unique capacity of replenishing the entire blood system. Under homeostatic conditions, HSCs are highly quiescent, rendering them resistant to cytotoxic stress and DNA damage, allowing them to maintain a healthy stem cell pool^1–5^. HSCs are typically recognized for their quiescent metabolic state and are primarily associated with glycolysis^6,7^. HSCs also sustain mitochondrial activity and exhibit flexibility in utilizing alternative metabolic pathways, including fatty acid oxidation, which can impact cellular lipid composition^8–10^.

Overall, lipids contribute to energy homeostasis as well as membrane structure and signalling^11,12^. While advances in genetic models and omics techniques have substantially expanded our understanding of HSC metabolism^13–18^, lipidomic studies—particularly in human HSCs—remain limited^13,19–24^. This is probably due to the scarcity of isolated HSCs, the limited availability of human BM samples and the challenges in developing accessible methods and workflows for such rare cell populations. Deciphering these metabolic cues is crucial for advancing HSC-based therapies, including long-term ex vivo expansion and novel treatments for haematological malignancies.

**Fig. 1:**
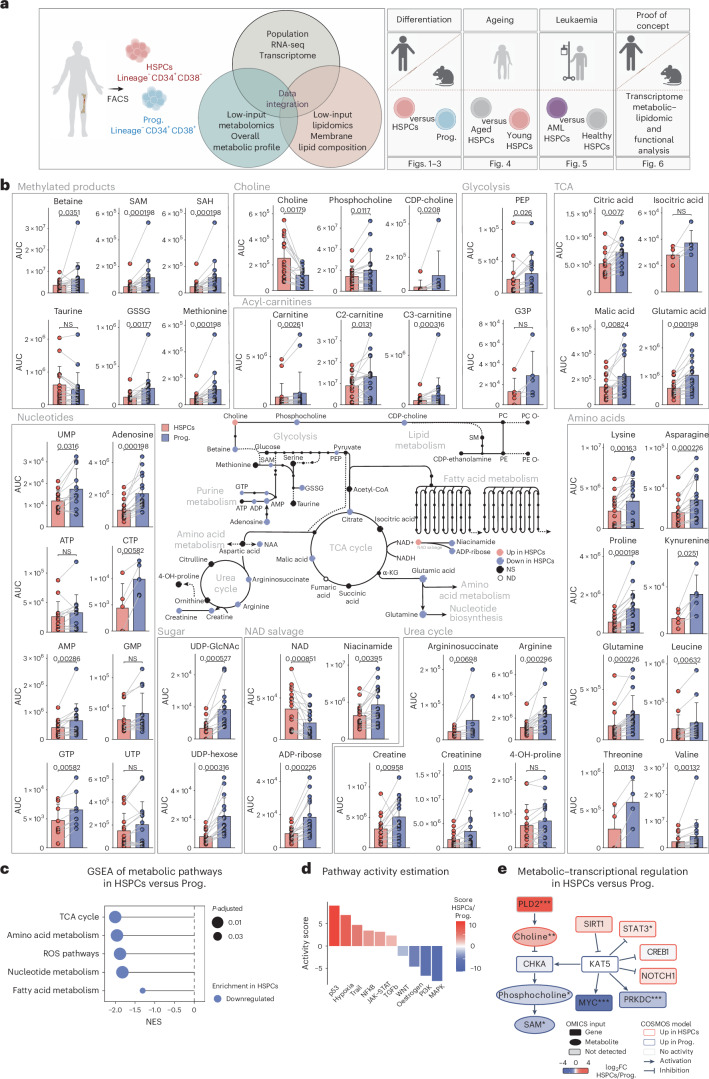
Metabolic profile of human BM HSPCs and their downstream progenitors. **a**, The experimental study design depicting the generated datasets and their respective figure numbers. Created with BioRender.com. **b**, A schematic summary of the metabolomics results per pathway in human HSPCs and progenitors. Quantification bar plots as mean ± standard deviation show raw intensity values per biological replicate. Paired samples are linked by grey lines. Differentially abundant metabolites are depicted as coloured dots in the scheme. *n* = 17. *P*-adjusted value after paired two-sided Student’s *t*-test or Wilcoxon test is shown. NS, not significant; ND, not detected. **c**, GSEA of metabolic pathways on RNA-seq of human HSPCs versus progenitors. *n* = 4. **d**, Pathway activity score estimated by decoupleR in HSPCs versus progenitors RNA-seq. Differentially activated pathways are shown (*P* < 0.05). **e**, A subnetwork representing the COSMOS mechanistic hypothesis of metabolic and transcriptomic regulation of choline pathway, based on HSPCs versus progenitors metabolomics and RNA-seq datasets. Each node represents a gene or metabolite. The border colour depicts the estimated activity by the model. The arrow shape corresponds to the type of regulation based on ground knowledge. The fill colour of elements shows their level of differential expression or abundance in our measured data. *P*-adjusted value; *P < 0.05, **P < 0.01, ***P < 0.001. In **b** and **c**, *n* indicates the number of biological replicates per condition. For **b**, ten independent experiments were performed. NAA, *N*-acetylaspartic acid; α-KG, alpha-ketoglutarate; PEP, 2-phosphoenolpyruvate; GSSG, glutathione disulfide.

Here, we sought to provide a comprehensive metabolic and lipidomic profile of human BM haematopoietic stem and progenitor cells (HSPCs, lineage^−^ CD34^+^ CD38^−^) by combining two low-input approaches: (1) targeted metabolomics for polar metabolites and (2) a newly optimized untargeted lipidomic workflow. Using 86 human BM samples throughout the study, we uncovered metabolic cues that are altered upon differentiation, ageing and acute myeloid leukaemia (AML) (Fig. 1a and Supplementary Table 1; data also available in an interactive platform at https://cabezas-lab.shinyapps.io/HumanMetabolomics/). We identified choline levels to be high in young healthy HSPCs, which declined upon ageing and decreased even further in leukaemia. As a proof of concept, we showed that supplementation with the metabolite choline enhances stemness, highlighting it as a regulator of HSPCs. Overall, this study serves as a distinct resource for investigating human HSC metabolism in the contexts of differentiation, ageing and leukaemia.

## Results

### Metabolic profile of human HSPCs and downstream progenitors

**Extended Data Fig. 1:**
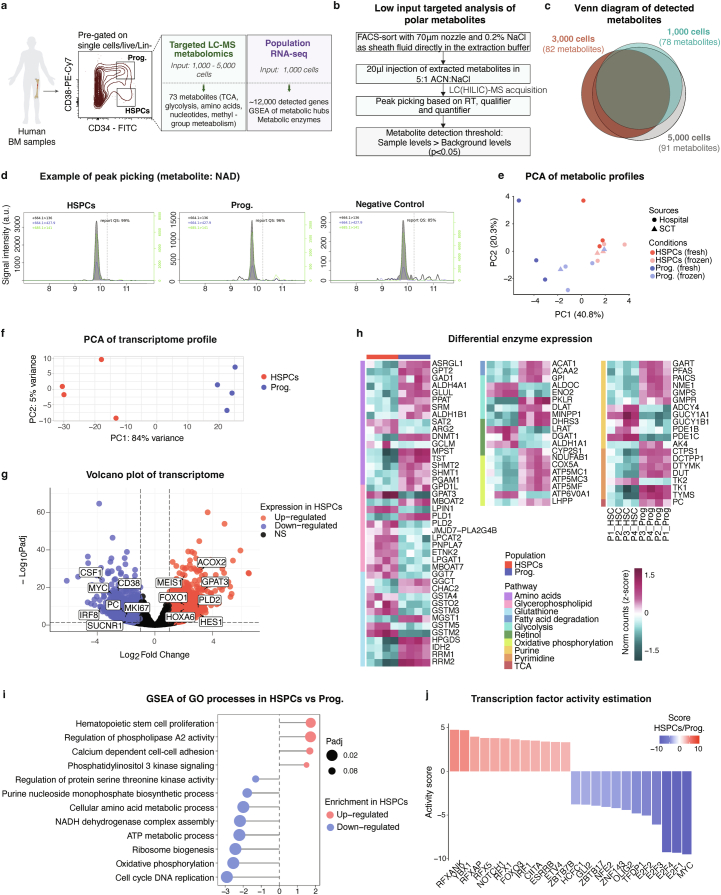
Metabolic profile of human BM HSPCs and their downstream progenitors. **a**, Experimental design and gating to characterize the targeted metabolomics and transcriptome of human HSPCs and progenitors. The input material per sample, number and type of detected metabolites and genes are indicated. **b**, Workflow of method and downstream analysis of low input targeted polar metabolomics in human HSPCs and progenitors. **c**, Venn diagram representing the detected metabolites above threshold with an input material of 1,000, 3,000, or 5,000 HSPCs. Number of metabolites per dataset is shown. n = 6. **d**, Representative example of metabolomics peaks in HSPCs, progenitors and background levels (Negative Control). Signal intensity was assessed by picking peaks with the same retention times as their respective qualifiers (green and blue lines). AUC, area under the curve. **e**, Metabolomics Principal component analysis (PCA) of HSPCs and progenitors isolated under different freezing conditions and from various sources as quality control. Conditions included samples with and without prior freezing; sources included commercially available samples (Stem Cell Technology; SCT) or hospital-derived samples. The analysis was based on significantly variable metabolites. n = 7. **f**, PCA of HSPCs and progenitors RNA-seq samples based on the top 500 most variable genes. n = 4. **g**, Volcano plot of differentially expressed genes (DEGs) in HSPCs vs progenitors RNA-seq. P-adjusted value. NS, not significant. **h**, Heatmap representing relative expression of differentially expressed KEGG enzymes in HSPCs vs progenitors (log2FC threshold = 1, p-adjusted < 0.05), classified in their respective metabolic pathways. **i**, Gene Set Enrichment Analysis (GSEA) of selected Gene Ontology (GO) processes on RNA-seq of HSPCs vs progenitors. P-adjusted value. NES, Normalized Enrichment Score. **j**, TF activity score estimated by decoupleR in HSPCs vs progenitors RNA-seq. Top 12 differentially activated TFs per condition are shown (p-value < 0.05). (**c**, **e**, **f**) n indicates the number of biological replicates per condition. For (**c**), 4 independent experiments were performed. For (**e**), 5 independent experiments were performed.

**Extended Data Fig. 2:**
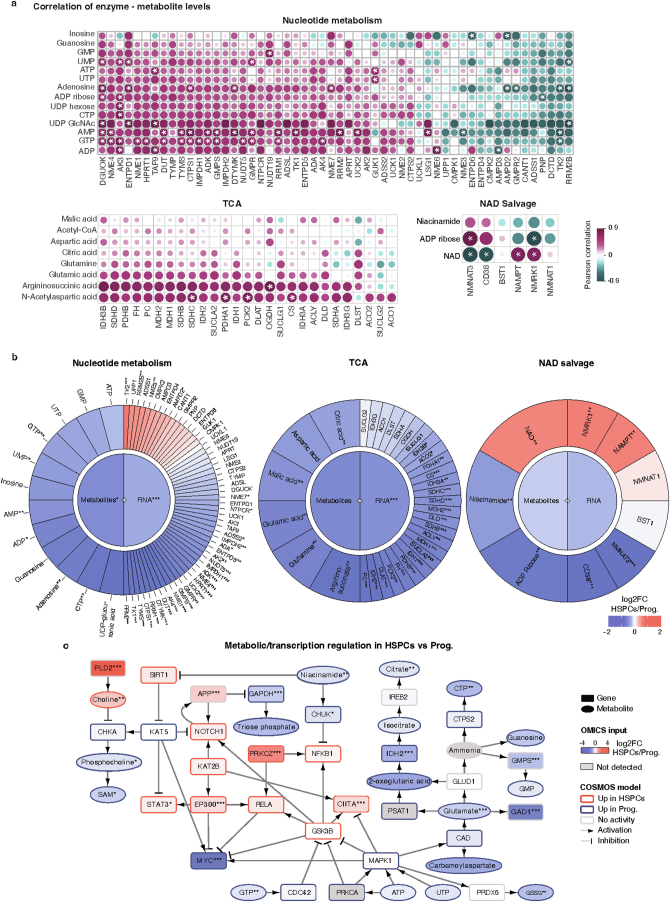
Metabolic profile of human BM HSPCs and their downstream progenitors. **a**, Pearson correlation between enzyme expression and metabolite abundance per sample in selected KEGG pathways. Color and size of the dots represent the correlation coefficients. n = 4. Correlation p-value; *p < 0.05. **b**, Metabolograms integrating metabolomics and transcriptomics data on selected KEGG pathways. Color represents the log2FC between HSPCs and progenitors. Inner circles indicate the average tendencies, and outer circles show individual genes/metabolites. P-adjusted value; *p < 0.05, **p < 0.01, ***p < 0.001. **c**, Subnetwork representing the COSMOS mechanistic hypotheses of metabolic/transcriptomic regulation of multiple connected pathways, based on HSPCs vs progenitors metabolomics and RNA-seq datasets. Each node represents a gene or metabolite. The border color depicts the estimated activity by the model. Arrow shape corresponds to the type of regulation based on ground knowledge. The fill color of elements shows their level of differential expression or abundance in our measured data. P-adjusted value; *p < 0.05, **p < 0.01, ***p < 0.001. (**a**) n indicates the number of biological replicates per condition.

To explore the metabolic landscape of human BM HSCs and identify potential stemness regulators, we isolated the HSC-enriched compartment (HSPCs; lineage^−^ CD34^+^ CD38^−^) and downstream progenitors (Prog.; lineage^−^ CD34^+^ CD38^+^) from young healthy individuals (20–40 years old) (Extended Data Fig. 1a). We utilized our previously established method for liquid chromatography–mass spectrometry (LC–MS)-based metabolomics on rare fluorescence-activated cell sorting (FACS)-sorted cell populations^16^ (Methods, Extended Data Fig. 1a,b and Supplementary Table 2), which allows the detection of polar metabolites such as tricarboxylic acid (TCA) cycle-related metabolites, amino acids and nucleotides. We first performed a titration experiment using 1,000, 3,000 and 5,000 human HSPCs (Extended Data Fig. 1c). Based on the number of detected metabolites and the low availability of HSPCs in each BM sample, we decided to proceed with 3,000 cells. Next, we generated and compared metabolomics data of human HSPCs and progenitors derived from multiple sources and from fresh or frozen BM material as a quality control. Principal component analysis (PCA) showed highly consistent metabolic profiles within populations independently of the sample source (Extended Data Fig. 1e and Supplementary Table 3). We next generated metabolomics data from HSPCs and progenitors isolated from 17 human healthy young BM donors. Seventy-three metabolites passed the stringent quality thresholds (Methods, Extended Data Fig. 1b,d and Supplementary Table 4) with 47 being significantly different between HSPCs and progenitors. TCA cycle-related metabolites such as citric acid, glutamic acid and malic acid were significantly less abundant in the HSPC compartment compared with progenitors (Fig. 1b). Nucleotide levels were lower in HSPCs, as well as the majority of amino acids and acyl-carnitines. We also found urea cycle metabolites to be significantly less abundant in HSPCs (for example, arginine, argininosuccinate and creatinine), with a concomitant reduction in the overall levels of redox-related metabolites, such as glutathione disulfide (Fig. 1b). Gene set enrichment analysis (GSEA) of the generated RNA sequencing (RNA-seq) data supported the metabolomics data as ‘TCA cycle’, ‘Amino acid metabolism’, ‘ROS pathway’, ‘Nucleotide metabolism’ and ‘Fatty acid metabolism’ pathways were downregulated in HSPCs (Fig. 1c, Extended Data Fig. 1f–i and Supplementary Table 4), suggesting that HSPCs are overall metabolically less active than their downstream progenitors, consistent with previous studies performed in mice^25–28^. Pathway and transcription factor (TF) activity was inferred from the transcriptomic data by decoupleR^29^ based on curated collections of pathway- and TF-target gene regulatory networks and linear modelling (Methods). Using this bioinformatic tool, we found the ‘Hypoxia’ pathway activated in HSPCs, in line with previous studies suggesting its critical role in inducing quiescent metabolic features^30^ (Fig. 1d). In addition, FOXO3, a TF known to maintain the redox balance in HSCs, was estimated to be active^31^ (Extended Data Fig. 1j). Meanwhile, JAK–STAT and PI3K signalling pathways as well as MYC and multiple TFs from the E2F family were found less active in HSPCs, in line with their higher quiescence (Fig. 1d and Extended Data Fig. 1j). To address the association between metabolites and their respective enzymes, we paired the transcriptome of each sample with their metabolic profiles. Overall, most enzyme–metabolite pairs showed a high correlation coefficient such as IDH3B–argininosuccinic acid from the TCA cycle (Extended Data Fig. 2a). We further integrated the omics data using metabolome–transcriptome plots (metabolograms)^32^ (Extended Data Fig. 2b). We observed significantly lower levels of both metabolites and enzymes associated with the TCA cycle and nucleotide metabolism in HSPCs, further indicating low metabolic activity.

Nicotinamide adenine dinucleotide (NAD) was found to be highly enriched in human HSPCs, while niacinamide and ADP-ribose were significantly more abundant in downstream progenitors (Fig. 1b). This is in line with lower expression of CD38 in human HSPCs, the enzyme responsible for converting NAD to ADP-ribose and niacinamide (Extended Data Fig. 2b). This observation suggests a reduced NAD salvage rate in HSPCs compared with more committed progenitors. Interestingly, our analysis revealed a high abundance of choline in the HSPC compartment (Fig. 1b). Choline can serve as a source of methyl groups through the production of betaine, or for the synthesis of key cell membrane lipids such as phosphatidylcholine (PC) and sphingomyelin (SM)^33^. In our dataset, the levels of betaine were lower in HSPCs, suggesting that choline might play a more critical role for the lipid production in HSCs. To gain further insight, we utilized a network modelling tool (Causal Oriented Search of Multi‐Omics Space; COSMOS) that infers a set of mechanistic hypotheses consistent with the data and existing knowledge by integrating our measured gene expression, metabolite abundance and TF activity with curated databases of regulatory interactions^34^. PLD2, an enzyme that catalyses the hydrolysis of phosphocholine into free choline, was highlighted by the model as a potential regulatory mechanism for the higher abundance of choline in HSPCs. In addition, SIRT1, an inhibitor of KAT5 TF, was predicted as another mechanism for choline accumulation in HSPCs, by blocking the enzyme CHKA from converting choline to phosphocholine (Fig. 1e and Extended Data Fig. 2c).

Taken together, we uncover unique metabolic cues of young human BM HSPCs and their downstream progenitors.

### Lipidomic profile of human HSPCs and downstream progenitors

**Fig. 2:**
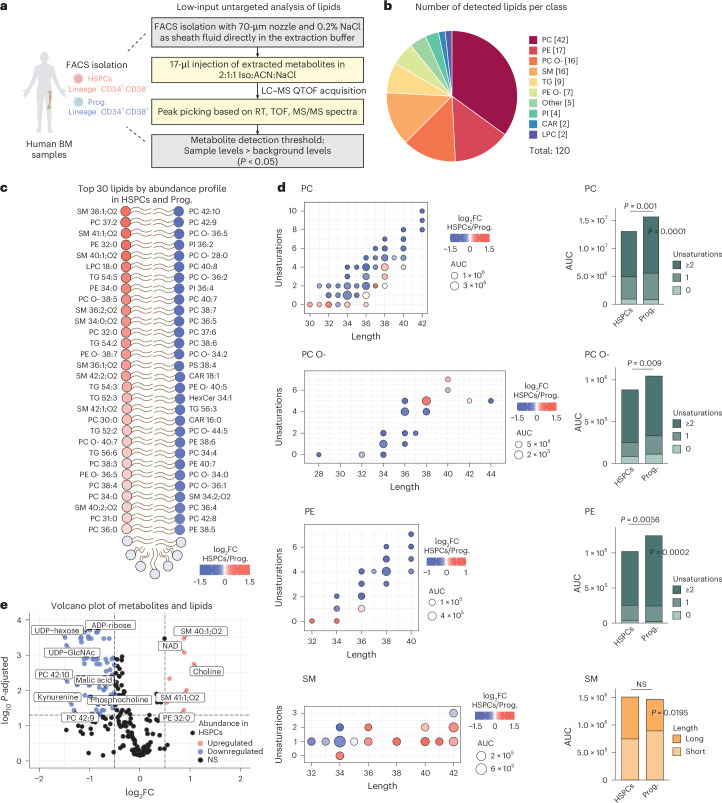
Lipid composition of human HSCs and their progenitors. **a**, The workflow of the method and analysis of low-input untargeted lipidomics in human HSPCs and progenitors. Created with BioRender.com. **b**, A pie chart depicting the number of detected lipids per class, with the total number per class indicated in square brackets. **c**, Lipheat depicting the top 30 detected lipids in HSPCs or progenitors ranked by log_2_FC value. *n* = 14. **d**, Left: dot plots depicting the total number of carbons (length) and double bonds (unsaturations) in the acyl chains of detected lipids, categorized by class. Colour shows the log_2_FC abundance in HSPCs versus progenitors. The dot size corresponds to the mean signal intensity of the detected peaks per lipid across all biological replicates. Right: bar plots of the summed signal intensity of lipids per class in HSPCs and progenitors. Subclasses were based on unsaturation levels (saturated (0 unsaturation), monounsaturated (1 unsaturation) and polyunsaturated (≥2 unsaturations)) or acyl-chain length (short (<36 carbons) and long (≥36 carbons)). Significantly different abundances per class and subclass are shown. Paired two-sided Student’s *t*-test or Wilcoxon test. **e**, A volcano plot of differentially abundant detected metabolites and lipids between HSPCs and progenitors. *P*-adjusted value after paired Student’s *t*-test or Wilcoxon test. NS, not significant. In **c**, *n* indicates the number of biological replicates per condition. For **b**–**d**, eight independent experiments were performed. Iso, isopropanol; CAR, carnitines; LPC, lyso-PC; PI, phosphatidylinositol; TG, triacylglyceride.

**Extended Data Fig. 3:**
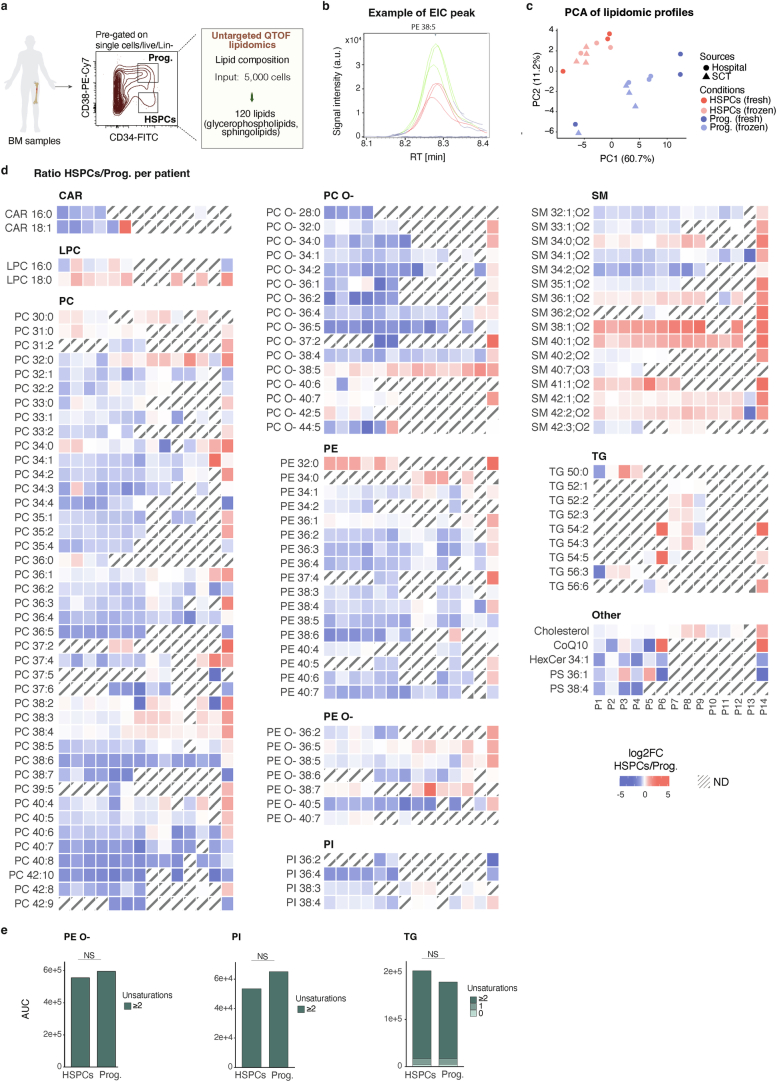
Lipid composition of human HSPCs and progenitors. **a**, Experimental design and gating to characterize the lipidome of human HSPCs and progenitors. The input material per sample, number and type of detected lipids are indicated. **b**, Example of extracted ion chromatogram (EIC) peak. **c**, Lipidomics PCA of HSPCs and progenitors isolated under different freezing conditions and from various sources as quality control. Conditions included samples with and without prior freezing; sources included commercially available samples (Stem Cell Technology; SCT) or hospital-derived samples. The analysis was based on significantly variable lipids. n = 9. **d**, Heatmap of all detected lipids passing quality thresholds in human untargeted lipidomics of HSPCs and progenitors. The log2FC values between paired HSPCs and progenitors per individual are depicted. n = 14. ND, not detected. **e**, Barplots of the summed signal intensity of lipids per class in HSPCs and progenitors. Subclasses were based on unsaturation levels [saturated (0 unsaturation), monounsaturated (1 unsaturation) and polyunsaturated (≥2 unsaturations)] or acyl-chain length [short (<36 carbons) and long (≥36 carbons)]. Significantly different abundances per class and subclass are shown. Paired two-sided Student’s t test or Wilcoxon test. NS, not significant; AUC, area under the curve. (**c**) n indicates the number of biological replicates per condition. For (**c**), 5 independent experiments were performed. For (**d**, **e**), 8 independent experiments were performed.

Next, we aimed to enhance our understanding of the metabolic profile of human BM HSCs by exploring their lipidome, which comprises vital membrane structure and signalling molecules not captured in our prior polar analysis. Therefore, we optimized a low-input untargeted lipidomics workflow by combining FACS with LC–MS analysis building upon our previously described low-input metabolomics method^16^. The LC–MS parameters used in this study are consistent with those in our previous work^34^, with differences outlined in the Methods. We FACS-sorted 5,000 HSPCs and downstream progenitors directly in a lipid extraction buffer (final composition: 50% 2-propanol, 25% acetonitrile and 25% water with 0.2% NaCl) and directly injected them in the LC–quadrupole time-of-flight (QTOF)-MS for non-targeted analysis of lipids (Methods, Fig. 2a and Extended Data Fig. 3a,b). As the analysis of polar metabolites has highlighted a potential role of choline in HSCs, we used positive ionization mode, which yields higher signal intensity for choline-containing lipids. As a quality control, we performed lipidomics of samples provided from various sources and from fresh and frozen BM material and found consistent lipidomic profiles ensuring the robustness of our method (Extended Data Fig. 3c and Supplementary Table 3). We then conducted a comprehensive analysis with a larger sample set (*n* = 14). A total of 120 lipids were detected in HSPCs and progenitors after applying our filtering criteria, mainly classified into: (1) glycerophospholipids (PC and phosphatidylethanolamine (PE) as well as ether-linked PC (PC O-) and ether-linked PE (PE O-)); (2) sphingolipids (SM) and (3) triacylglycerides (TG) (Fig. 2b, Extended Data Fig. 3d and Supplementary Table 4).

**Extended Data Fig. 4:**
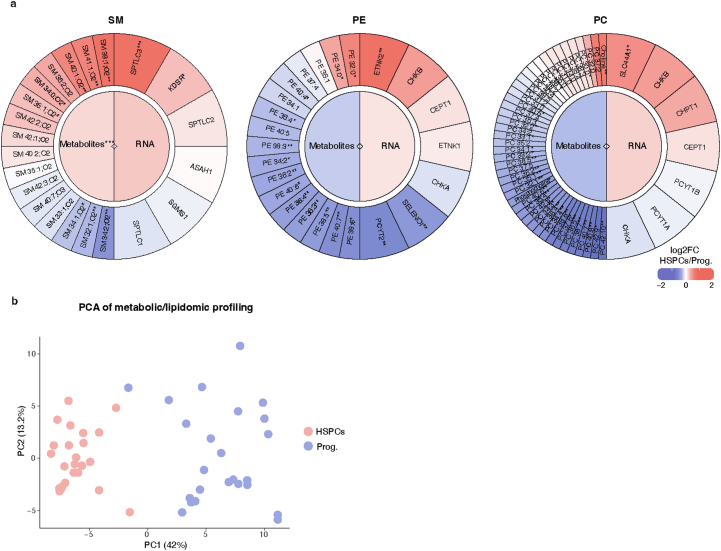
Lipid composition of human HSPCs and progenitors. **a**, Metabolograms integrating metabolomics/lipidomics and transcriptomics data on selected KEGG pathways or modules. Color represents the log2FC between HSPCs and progenitors. Inner circles indicate the average tendencies, and outer circles show individual genes and metabolites/lipids. P-adjusted value; *p < 0.05, **p < 0.01, ***p < 0.001. **b**, PCA of HSPCs and progenitors based on metabolomics and lipidomics. The analysis was based on significantly variable metabolites and lipids. n = 24. NS, not significant. (**b**) n indicates the number of biological replicates per condition.

We initially ranked all lipids according to fold change differences between HSPCs and downstream progenitors and found that the most enriched lipid species in HSPCs belonged to SM (Fig. 2c and Extended Data Fig. 3d). Next, we visualized individual lipids on the basis of acyl chain length and unsaturation degree (number of double bonds) and assessed potential differences by comparing the total signal intensity of lipids per class and subclass (Fig. 2d and Extended Data Fig. 3e). We observed that most PC, PC O- and PE were more abundant in the progenitor compartment, driven mainly by their polyunsaturated fraction (≥2 double bounds), while the long-chain SM fraction (≥36 carbons) was significantly enriched in the HSPC compartment. Metabologram plots revealed that enzymes involved in SM and sphingosine synthesis were notably enriched in HSPCs, whereas PE species and specific enzymes important for their synthesis were upregulated in the progenitor compartment (Extended Data Fig. 4a).

In summary, we have optimized a low-input untargeted lipidomics workflow, which together with our targeted polar analysis reveals that HSPCs have distinct metabolic and lipidomic profiles compared with their downstream progenitors (Figs. 1 and 2e and Extended Data Fig. 4b). Specifically, HSPCs show higher levels of long-chain SM and lower levels of polyunsaturated PC and PE relative to more differentiated progenitors, suggesting that they display distinct membrane characteristics, such as fluidity or permeability.

### Mouse HSC metabolic profile resembles human HSCs

**Fig. 3:**
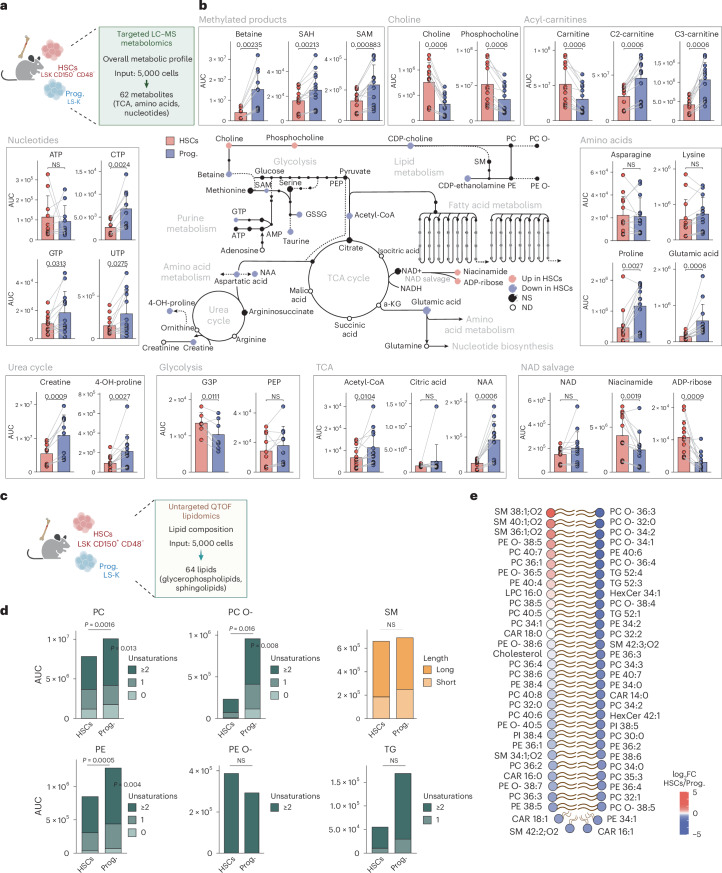
Metabolic and lipidomic profile of mouse HSCs and progenitors. **a**, The experimental design to characterize the metabolome of mouse HSCs and progenitors. The input material per sample, number and type of detected metabolites and genes are indicated. **b**, A schematic summary of metabolomics results per pathway in mouse HSCs and progenitors. Quantification bar plots as mean ± standard deviation show raw values per biological replicate. Paired samples are linked by grey lines. Differentially abundant metabolites are depicted as coloured dots in the scheme. *n* = 13. *P*-adjusted value after paired two-sided Student’s *t*-test or Wilcoxon test is shown. **c**, The experimental design to characterize the lipidome of mouse HSCs and progenitors. The input material per sample, number and type of detected lipids are indicated. **d**, Bar plots of the summed signal intensity of lipids per class in HSCs and progenitors. Subclasses were based on unsaturation levels or acyl-chain length. Significantly different abundances per class and subclass are shown. Paired two-sided Student’s *t*-test or Wilcoxon test. **e**, Lipheat depicting the detected lipids in HSCs versus progenitors ranked by log_2_FC value. *n* = 10. In **b** and **d**, *n* indicates the number of biological replicates per condition. For **b**, four independent experiments were performed. For **d** and **e**, three independent experiments were performed. Panels **a** and **c** created with BioRender.com.

**Extended Data Fig. 5:**
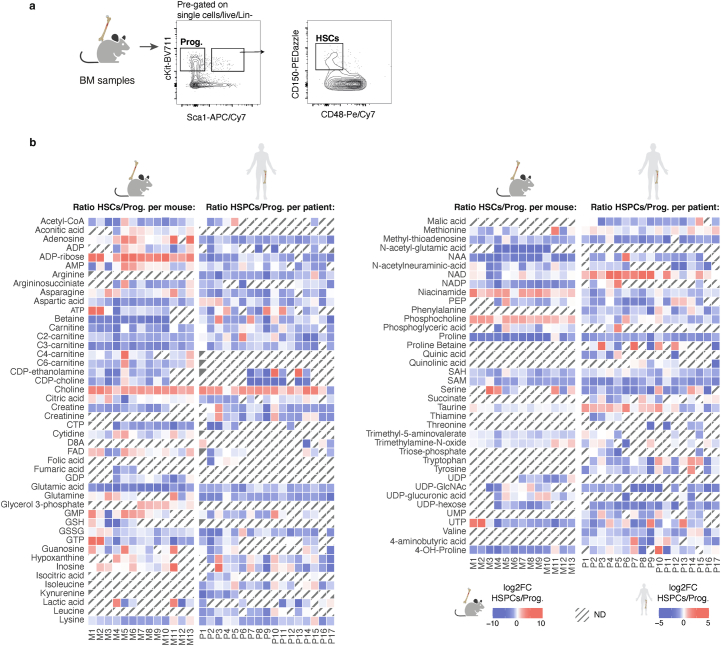
Metabolic and lipidomic profile of mouse HSCs and progenitors. **a**, Representative FACS gating scheme of mouse HSCs (LSK CD150+ CD48-) and progenitors (LS-K). **b**, Heatmap of all detected metabolites passing quality thresholds in both mouse and human targeted metabolomics of HSCs/HSPCs and progenitors. The log2FC values between paired HSCs/HSPCs and progenitors per individual are depicted. n = 13 and 17 for mouse and human, respectively. (**b**) n indicates the number of biological replicates per condition.

To examine whether the observed metabolic profiles in human HSCs are conserved in mice, we performed metabolomics and lipidomics on the equivalent mouse BM HSC-enriched (lineage^−^ Sca1^+^ cKit^+^ (LSK) CD150^+^ CD48^−^) and downstream progenitor (lineage^−^ Sca1− cKit^+^ (LS-K)) populations (Fig. 3a, Extended Data Fig. 5a and Supplementary Table 5). Similar to human HSPCs, metabolites from main pathways such as TCA cycle, amino acids, nucleotides and acyl-carnitines were significantly less abundant in mouse HSCs compared with progenitors (Fig. 3b and Extended Data Fig. 5b). Interestingly, choline was also more abundant in mouse HSCs, suggesting a potential critical role for HSC stemness in both species. By contrast, the NAD salvage metabolites, including NAD, ADP-ribose and niacinamide, displayed rather opposite profiles between mouse and human HSCs. These differences might be explained by the differential expression of the enzyme CD38 (ref. ^35^).

Our lipidomics analysis also revealed numerous similarities between mouse and human, as most of the lipid classes (PC, PC O- and PE) were significantly less abundant in mouse HSCs compared with their downstream progenitors (Fig. 3c,d). We also observed the same three long-chain SM species highly enriched in mouse and human HSCs and HSPCs, namely SM 38:1;O2, SM 40:1;O2 and SM 36:1;O2 (Fig. 3e).

Collectively, our findings demonstrate highly similar metabolic profiles between human and mouse HSCs and HSPCs (Extended Data Fig. 5b), including an enrichment of choline and certain long-chain SM in HSCs.

### Human HSPCs show an altered metabolic profile upon ageing

**Fig. 4:**
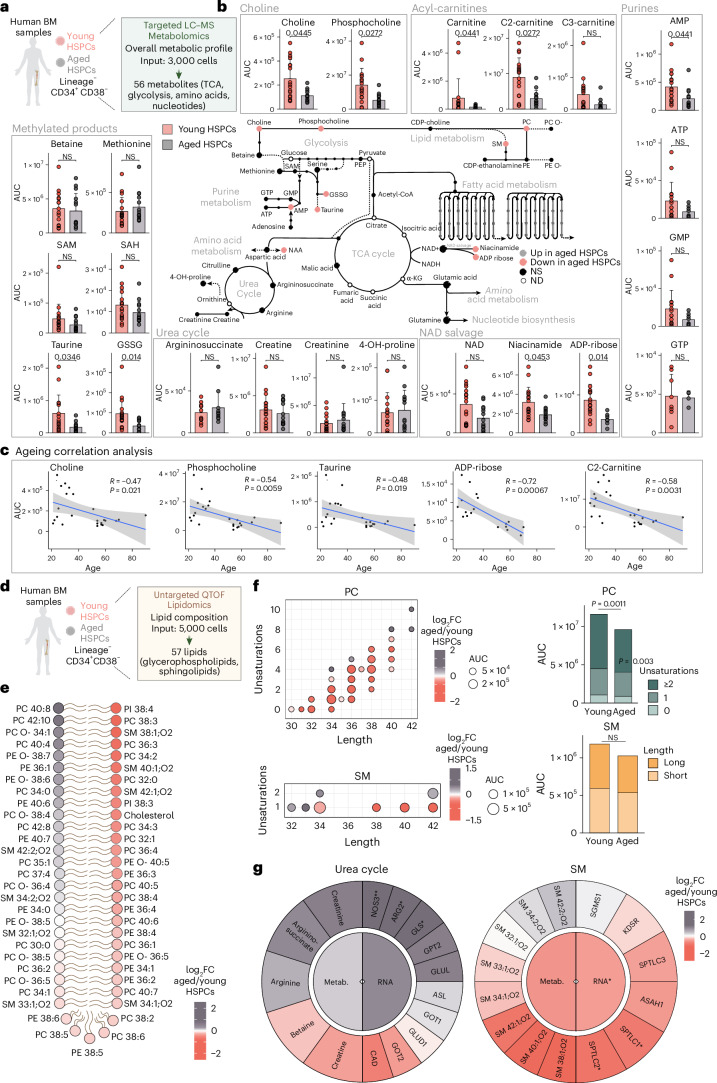
Metabolic and lipidomic profile of human HSPCs upon ageing. **a**, The experimental design to characterize the metabolome of aged and young human HSPCs. **b**, A schematic summary of metabolomics results per pathway in human young and aged HSPCs. Quantification bar plots as mean ± standard deviation show raw values per biological replicate. Differentially abundant metabolites are depicted as coloured dots in the scheme. *n* = 14–17. *P*-adjusted value after unpaired two-sided Student’s *t*-test or Wilcoxon test is shown. **c**, Pearson correlation between metabolite abundance and age in patients in which these data were provided. Linear model, correlation coefficient and *P* value are depicted. **d**, The experimental design to characterize the lipidome of aged and young human HSPCs. **e**, Lipheat depicting the detected lipids in HSPCs versus progenitors ranked by log_2_FC value. *n* = 6–10. **f**, Left: dot plots depicting the total number of carbons (length) and double bonds (unsaturations) in the acyl chains of detected lipids, categorized by class. Colour shows the log_2_FC abundance in aged versus young HSPCs. Dot size corresponds to the mean signal intensity of the detected peaks per lipid across all biological replicates. Right: bar plots of the summed signal intensity of lipids per class in aged and young HSPCs. Subclasses were based on unsaturation levels or acyl-chain length. Significantly different abundances per class and subclass are shown. Paired two-sided Student’s *t*-test or Wilcoxon test. **g**, Metabolograms integrating metabolomics, lipidomics and transcriptomics data on selected KEGG pathways or modules. Colour represents the log_2_FC between aged and young HSPCs. Inner circles indicate the average tendencies, and outer circles show individual genes or metabolites and lipids. *P*-adjusted value; *P < 0.05, **P < 0.01, ***P < 0.001. In **b** and **e**, *n* indicates the number of biological replicates per condition. For **b**, 13 independent experiments were performed. For **e** and **f**, nine independent experiments were performed. Panels **a** and **d** created with BioRender.com.

**Extended Data Fig. 6:**
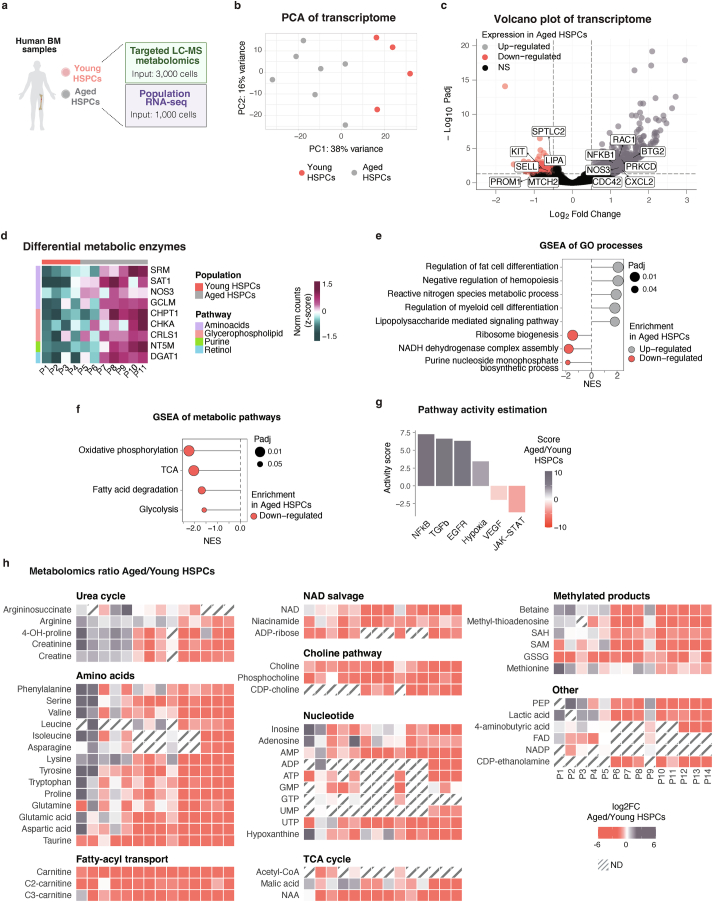
Metabolic and lipidomic profile of human HSPCs upon aging. **a**, Experimental design to characterize the metabolome and transcriptome of young and aged human HSPCs. The input material per sample is indicated. **b**, PCA of young and aged HSPCs RNA-seq samples based on the top 500 most variable genes. n = 4–7. **c**, Volcano plot of transcriptome DEGs in young vs aged HSPCs. P-adjusted value. NS, not significant. **d**, Heatmap representing relative expression of differentially expressed KEGG enzymes in young vs aged HSPCs (log2FC threshold = 1, p-adjusted < 0.05), classified in their respective metabolic pathways. **e**, GSEA of selected GO processes on RNA-seq of aged vs young HSPCs. P-adjusted value. **f**, GSEA of selected metabolic pathways on RNA-seq of aged vs young HSPCs. P-adjusted value. **g**, Pathway activity score estimated by decoupleR in young vs aged HSPCs RNA-seq. Differentially activated pathways are shown (p-value < 0.05). **h**, Heatmap of all detected metabolites passing quality thresholds in human targeted metabolomics of aged and young HSPCs. The log2FC values between each aged biological replicate and the average of young HSPCs are depicted. n = 14–17. ND, not detected. (**b**, **h**) n indicates the number of biological replicates per condition. For (**h**), 13 independent experiments were performed.

We next aimed to assess the metabolic features of human HSPCs upon ageing. To this end, we performed metabolomic, lipidomic and transcriptomic analyses on HSPCs that were isolated from aged (50–90 years old) and young (20–40 years old) individuals (Fig. 4a, Extended Data Fig. 6a and Supplementary Table 6). GSEA analysis of the transcriptome highlighted terms such as ‘ROS metabolic process’, ‘Regulation of myeloid cell differentiation’ and ‘Regulation of fat cell differentiation’ to be enriched in aged HSPCs in agreement with recent studies^36–39^ (Extended Data Fig. 6b–g).

The combination of targeted metabolomics and untargeted lipidomics enabled the detection of 56 polar metabolites and 57 lipids (Fig. 4a–e). Creatinine and argininosuccinic acid, as well as enzymes involved in the urea cycle, were slightly more abundant in aged HSPCs, suggesting that the urea pathway is upregulated upon ageing (Fig. 4b,g and Extended Data Fig. 6h). Meanwhile, carnitine and acyl-carnitines, which are involved in mitochondrial fatty oxidation, were reduced upon ageing, and supported by the downregulated GSEA term ‘Fatty acid degradation’ (Extended Data Fig. 6f). The NAD salvage pathway metabolites ADP-ribose and niacinamide were significantly lower in aged HSPCs, in agreement with the beneficial effects observed by boosting NAD levels upon ageing in HSCs^40^. Taurine levels were reduced in aged HSPCs, further supporting the previously proposed role of taurine deficiency as a systemic driver of ageing^41^ (Fig. 4b and Extended Data Fig. 6h). To gain additional insights, we conducted correlation analyses across the full age range of our human samples, revealing age-related changes in specific metabolites such as ADP-ribose, taurine, *N*-acetylaspartic acid and carnitine C2, supporting continuous changes in metabolite levels upon HSPC ageing (Fig. 4c).

**Extended Data Fig. 7:**
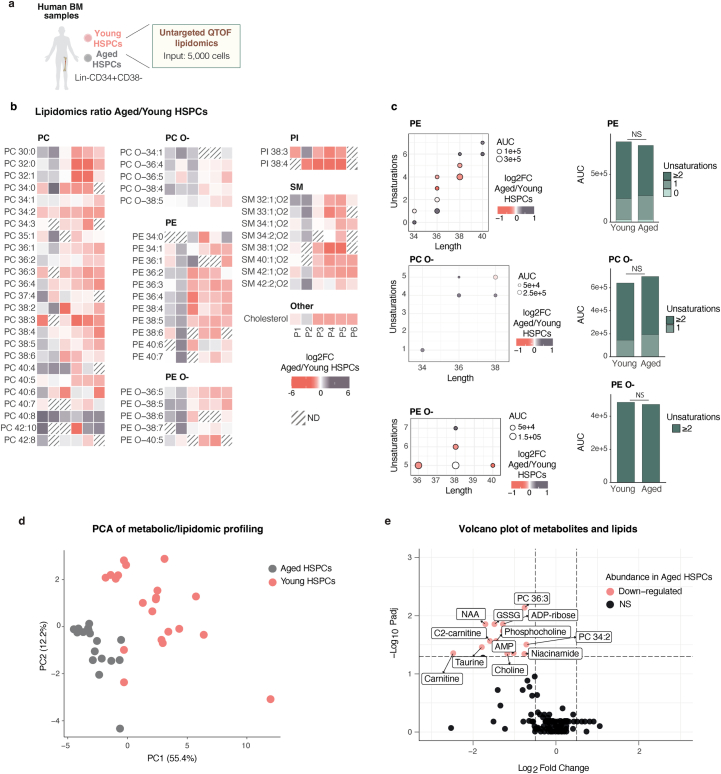
Metabolic and lipidomic profile of human HSPCs upon aging. **a**, Experimental design to characterize the lipidome of young and aged human HSCs. The input material per sample is indicated. **b**, Heatmap of all detected lipids passing quality thresholds in untargeted lipidomics of human aged and young HSPCs. The log2FC values between each aged biological replicate and the average of young HSPCs are depicted. n = 6–10. ND, not detected. **c**, Left, dotplots depicting the total number of carbons (length) and double bonds (unsaturations) in the acyl-chains of detected lipids, categorized by class. Color shows the log2FC abundance in aged vs young HSPCs. Dot size corresponds to the mean signal intensity of the detected peaks per lipid across all biological replicates. Right, barplots of the summed signal intensity of lipids per class in aged and young HSPCs. Subclasses were based on unsaturation levels or acyl-chain length. Differential abundances per class and subclass are shown. Paired two-sided Student’s t test or Wilcoxon test. NS, not significant; AUC, area under the curve. **d**, PCA of aged and young HSPCs based on metabolomics and lipidomics. The analysis was based on significantly variable metabolites and lipids. n = 14–17. **e**, Volcano plot of differentially abundant metabolites and lipids in aged and young HSPCs. P-adjusted value. NS, not significant. (**b**, **d**) n indicates the number of biological replicates per condition. For (**b**, **c**), 8 independent experiments were performed.

Choline and phosphocholine were also significantly lower in aged HSPCs, and correlation analysis confirmed this trend across the age range, whereas betaine levels remained unchanged (Fig. 4b,c and Extended Data Fig. 6h). The reduction of choline was accompanied by an altered lipid composition, as some choline-headed lipids, particularly PC, were reduced upon ageing (Fig. 4d–f and Extended Data Fig. 7a–c). In addition, aged HSPCs showed a reduction of polyunsaturated PC together with a tendency for reduced levels of long-chain SM (Fig. 4f). Our transcriptome data also showed that enzymes involved in SM and sphingosine synthesis were downregulated in aged HSPCs (Fig. 4g). These findings demonstrate that the lipid composition, particularly the choline-headed lipid fraction, is altered upon ageing in HSPCs (Extended Data Fig. 7d,e).

### Human HSPCs display metabolic alterations in patients with AML

**Fig. 5:**
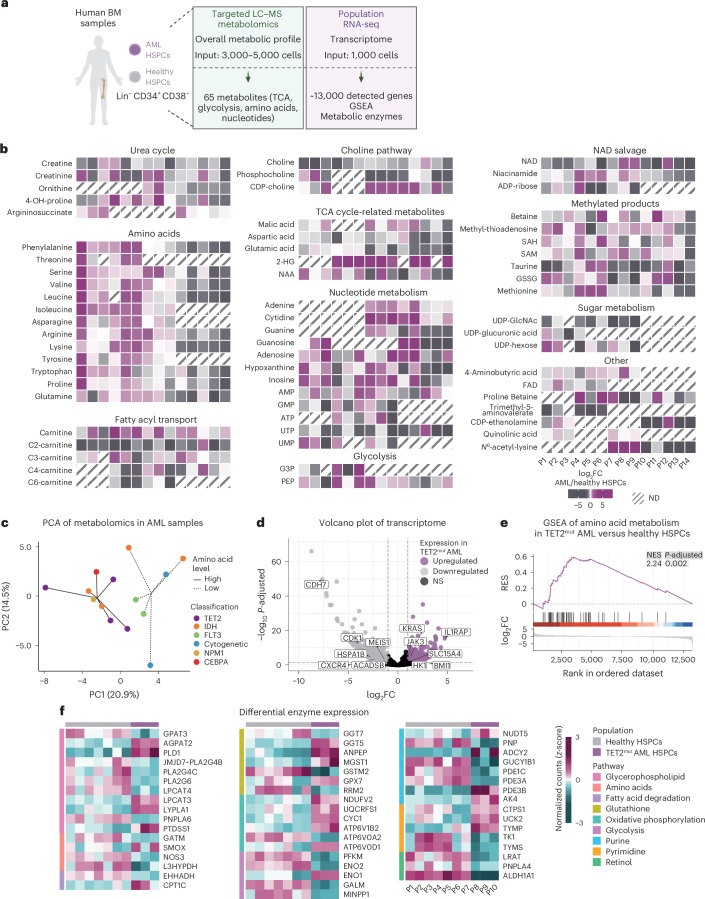
Metabolic profile of AML HSPCs. **a**, The experimental design to characterize the metabolome and transcriptome of HSPCs (lineage^−^ CD34^+^ CD38^−^) from healthy participants and patients with AML. The input material per sample and number of detected metabolites and genes are indicated. Created with BioRender.com. **b**, A heat map of all detected metabolites passing quality thresholds in human targeted metabolomics of AML and healthy HSPCs. The log_2_FC values between each AML biological replicate and the average of healthy HSPCs are depicted. *n* = 15–17; 2-HG, 2-hydroxyglutarate. **c**, PCA of AML HSPC samples normalized to their healthy counterparts based on all detected metabolites. Colour depicts the categorization of patients based on their common mutations. Lines represent two groups based on the amino acids levels compared with healthy participants. **d**, A volcano plot of transcriptome differentially expressed genes (DEGs) in TET2-mutated AML versus healthy HSPCs. *P*-adjusted value. *n* = 3–7. **e**, GSEA of cellular amino acid metabolic process GO term in TET2-mutated AML versus healthy HSPC RNA-seq. *P*-adjusted value. **f**, A heat map representing the relative expression of differentially expressed KEGG enzymes in TET2-mutated AML versus healthy HSPCs (log_2_FC threshold of 1, *P*-adjusted < 0.05), classified in their respective metabolic pathways. In **b**–**d**, *n* indicates the number of biological replicates per condition. For **b**, 12 independent experiments were performed. PC, principal component.

**Extended Data Fig. 8:**
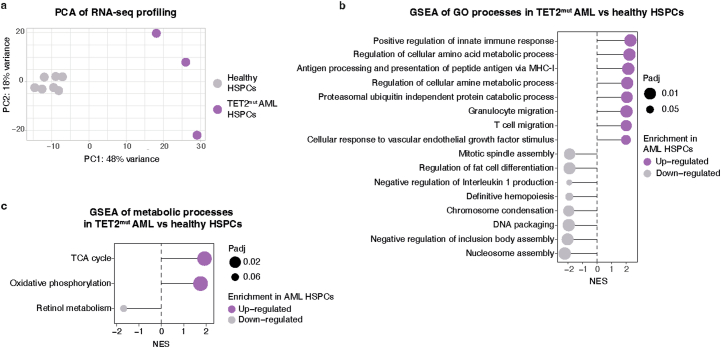
Metabolic profile of AML-HSPCs. **a**, PCA of AML and healthy HSPCs RNA-seq samples based on the top 500 most variable genes. n = 3–7. **b**, GSEA of GO processes on RNA-seq of TET2 mutated AML vs healthy HSPCs. P-adjusted value. **c**, GSEA of selected metabolic processes on RNA-seq of TET2 mutated AML vs healthy HSPCs. P-adjusted value. (**a**) n indicates the number of biological replicates per condition.

AML is the most common acute leukaemia in adults^42^, and metabolic alterations constitute a distinct feature of leukaemic stem cells and blasts^17,43^. Therefore, we sought to compare HSPCs isolated from patients with AML with those of age-matched healthy individuals by performing our combined analysis for low-input metabolomics and transcriptomics (Fig. 5a and Supplementary Table 7). Choline levels were lower across AML samples, independently of their diverse cytogenetic and molecular profiles, further highlighting its potential role as HSC regulator. 2-Hydroxyglutarate, a known onco-metabolite frequently elevated in AML, was also found to be higher in the majority of our AML samples (Fig. 5b). Although the genetic mutations across these AML samples are highly diverse, we observed two distinct metabolic groups driven by amino acid abundance in comparison with their healthy HSPC counterparts (Fig. 5b,c). Interestingly, samples with TET2 mutations were exclusively found in the high-abundance cluster, while those with FLT3 mutations appeared predominantly in the low-abundance group, with other genotypes being distributed across both clusters. GSEA from TET2-mutated samples supported this finding as ‘Regulation of cellular amino acid metabolic process’ was upregulated in AML HSPCs (Fig. 5d–f and Extended Data Fig. 8a–c) suggesting a distinct role of amino acids in specific leukaemias.

Despite the diverse cytogenetic and molecular profiles of the AML samples analysed, we observed distinct metabolic profiles, such as amino acid abundance being linked to specific genetic mutations. Meanwhile, we identified a consistent reduction of choline across the majority of AML samples (13 out of 14 samples), highlighting its potential role as an HSC regulator.

### Choline treatment preserves HSC function via membrane lipids

Based on the observation that choline levels are consistently higher in both human and mouse HSCs and HSPCs compared with progenitors, while reduced in HSPCs derived from aged patients and patients with AML, we decided to investigate its role in regulating HSCs.

**Fig. 6:**
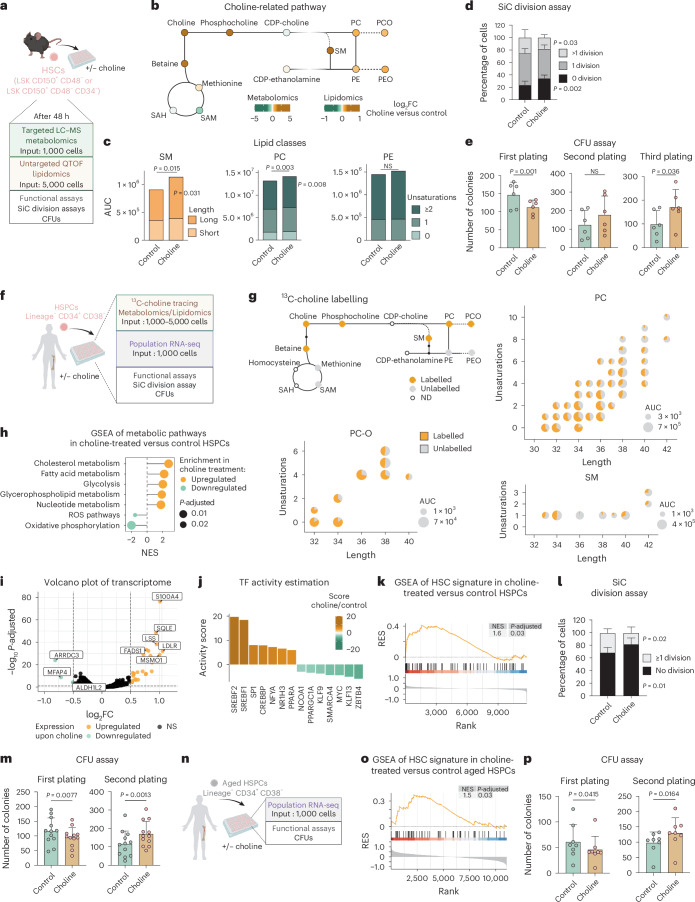
Choline supplementation fuels lipid composition and preserves HSPC function. **a**, The experimental design to characterize the effect of choline on mouse HSCs. HSCs were treated with 5 mM choline (or control treatment) for 48 h before functional analysis or subsequently FACS sorted for metabolomics and lipidomics. **b**, A schematic summary illustrating the detected metabolites and lipid classes in choline-treated versus control HSCs. Coloured dots represent log_2_FC values. *n* = 6. **c**, Bar plots of the summed signal intensity of lipids per class in choline-treated and control HSCs. Subclasses were based on unsaturation levels or acyl-chain length. Significantly different abundances per class and subclass are shown. Paired two-sided Student’s *t*-test or Wilcoxon test. **d**, Single-cell (SiC) division assay after 48 h in vitro treatment of mouse HSCs with 5 mM choline (or control treatment). The percentage of cells is shown. *n* = 11. **e**, CFU assay of mouse HSCs treated with 5 mM choline (or control treatment). The total number of colonies is depicted. *n* = 6. **f**, The experimental design to characterize the effect of choline on human HSPCs. Human HSPCs were treated with 10 mM choline (or control treatment) for 48 h before functional analysis or subsequently FACS sorted for population RNA-seq and metabolomics and lipidomics after stable isotope labelling. **g**, A schematic representation depicting the incorporation of ^13^C-labelled choline in metabolomics and lipidomics after 72 h treatment in human HSPCs. Pie charts show the ratio of ^13^C-labelled lipid species per class based on unsaturation levels and acyl-chain length. *n* = 6. **h**, GSEA of selected metabolic pathways in RNA-seq of choline-treated versus control HSPCs. *n* = 3. **i**, A volcano plot of transcriptome DEGs in choline-treated versus control HSPCs. **j**, The TF activity score estimated by decoupleR in choline-treated versus control HSPC RNA-seq. The top seven differentially activated pathways per condition are shown (*P* < 0.05). **k**, The GSEA profile of HSC signature in choline-treated versus control HSPC RNA-seq. **l**, SiC division assay after 48 h in vitro treatment of human HSPCs with 10 mM choline (or control treatment). The percentage of cells is shown. *n* = 6. **m**, CFU assay of human HSPCs treated with 10 mM choline (or control treatment). The total number of colonies is depicted. *n* = 12. **n**, The experimental design to characterize the effect of choline on aged human HSPCs. HSPCs were treated with 10 mM choline (or control treatment) for 48 h before functional analysis or population RNA-seq. **o**, GSEA profile of HSC signature in choline-treated versus control aged HSPCs RNA-seq. *n* = 3. **p**, CFU assay of human aged HSPCs treated with 10 mM choline (or control treatment). The total number of colonies is depicted. *n* = 8. In **d**, **e**, **l**, **m** and **p**, data are presented as mean ± s.d. For **d** and **l**, statistical significance was determined using two-way ANOVA; for **e**, **m** and **p** statistical significance was determined using paired Student’s *t*-test. *n* indicates the number of biological replicates per condition. For **b**, **c** and **g**, two independent experiments were performed. For **m** and **p**, five and four independent experiments were performed, respectively. Panels **a**, **f** and **n** created with BioRender.com. Source data

**Extended Data Fig. 9:**
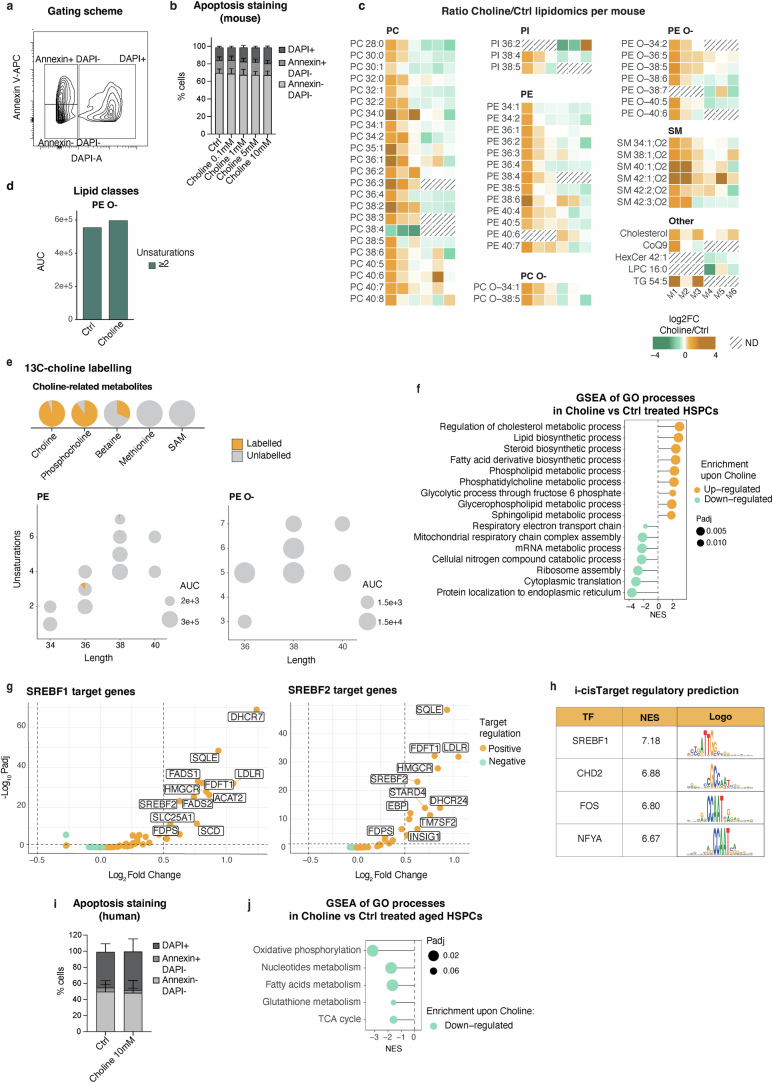
Choline supplementation modulates lipid composition and preserves HSPC function. **a**, Gating scheme for apoptosis quantification by Annexin V and DAPI staining. **b**, Apoptosis quantification in mouse HSCs after 48 h choline treatment using Annexin V and DAPI staining. **c**, Heatmap of all detected lipids passing quality thresholds in untargeted lipidomics of choline-treated and control HSCs. The log2FC values between paired choline-treated and control HSCs per individual are depicted. n = 6. **d**, Barplots of the summed signal intensity of lipids per class in choline-treated and control HSCs. Subclasses were based on unsaturation levels or acyl-chain length. Paired two-sided Student’s t test or Wilcoxon test. **e**, Representation of incorporation of 13C-labelled choline in metabolomics and lipidomics after 72 h treatment in human HSPCs. Pie charts depict the ratio of 13C-labelled metabolites or lipid species per class based on unsaturation levels and acyl-chain length. Size in lipid pie charts represents signal intensity. n = 6. AUC, area under the curve. **f**, GSEA of selected GO processes on choline vs control treated human HSPCs. P-adjusted value. n = 3. **g**, Volcano plot of transcriptome depicting SREBF1 (left) and SREBF2 (right) target genes in choline vs control treated HSPCs by decoupleR. Color shows the regulation of targets by the respective TF. P-adjusted value. **h**, i-cisTarget prediction of TF regulatory motifs in up-regulated genes in choline vs control treated human HSPCs. TF logo is depicted. NES, Normalized Enrichment Score. **i**, Apoptosis quantification in human CD34+ cells after 48 h choline treatment using Annexin V and DAPI staining. **j**, GSEA of selected GO processes on choline vs control treated human aged HSPCs. P-adjusted value. n = 3. (**b**) Data are presented as mean ± SD. (**c**, **e**, **f** and **i**) n indicates the number of biological replicates per condition. For (**c**–**e**), 2 independent experiments were performed. Source data

First, we treated mouse HSCs in vitro with choline and performed LC–MS-based targeted polar metabolomics (Fig. 6a, Extended Data Fig. 9a,b and Supplementary Table 8). We observed elevated choline and phosphocholine levels after treatment, indicating that HSCs can uptake the metabolite. Betaine, one of the downstream metabolites of choline, was also higher; however, this was not accompanied by any increase in methylated products (such as *S*-adenosyl-methionine (SAM) and methionine), suggesting that this pathway is not drastically affected (Fig. 6b). Given that choline can also be used as a head group to produce phospholipids, we determined the lipid composition in HSCs after treatment by untargeted lipidomics (Fig. 6c, Extended Data Fig. 9c,d and Supplementary Table 8). PC and SM, both choline-headed lipids, were significantly more abundant after treatment (Extended Data Fig. 6d). No differences were observed in the overall levels of PE and PE O- after treatment. We next explored whether choline regulates mouse HSC function. In vitro single-cell division assays of HSCs revealed an enhanced quiescent state upon choline treatment (Fig. 6d). Serial colony-forming unit (CFU) assays showed enhanced in vitro self-renewal capacity after choline treatment, as indicated by the elevated number of colonies produced (Fig. 6e; see third plating).

To investigate choline’s metabolic fate in human HSPCs, we performed stable isotope labelling with ^13^C-choline for 72 h in vitro and subsequently performed metabolomics and lipidomics (Fig. 6f,g). We observed significant incorporation of choline into PC, PC O- and SM, indicating its primary role in lipid biosynthesis. As expected, no significant incorporation of heavy carbons was observed in PE and PE O- after treatment (Extended Data Fig. 9e). While we detected minor ^13^C-labelling of betaine, no labelling was observed in downstream one-carbon metabolites such as methionine and SAM. This suggests that choline’s function in HSPCs is largely limited to lipid production rather than one-carbon metabolism, consistent with the findings in the mouse.

To gain additional mechanistic insights, we conducted population RNA-seq on human HSPCs treated with choline for 72 h. Pathway enrichment analysis showed a significant upregulation of lipid biosynthetic processes, including ‘Lipid biosynthetic process’, ‘Phospholipid metabolic process’ and ‘Sphingolipid metabolic process’, further corroborating our ^13^C-labelling results (Fig. 6h,i and Extended Data Fig. 9f). Using the decoupleR framework to infer TF activity from our transcriptomic data, we identified increased activity of SREBF1 and SREBF2, two sterol regulatory element-binding proteins (SREBPs) essential for cholesterol and fatty acid metabolism, in response to choline treatment^44^ (Fig. 6j and Extended Data Fig. 9g,h). In addition, choline supplementation promoted an enhanced HSC quiescent molecular signature, suggesting a beneficial effect on human HSPCs (Fig. 6k). To evaluate the functional impact of choline on human HSPCs, we performed in vitro single-cell division experiments and serial CFU assays. We observed that, in the presence of choline, human HSPCs show enhanced quiescence and in vitro self-renewal capacity (Fig. 6k–m and Extended Data Fig. 9i). Of note, aged HSPCs treated with choline show enhanced in vitro self-renewal capacity as well as the maintenance of an HSC molecular signature, further highlighting the potential of choline supplementation to preserve HSC function upon ageing (Fig. 6n–p and Extended Data Fig. 9j).

Together, our findings show that choline supplementation affects the lipidomic profile of mouse and human HSPCs and preserves their quiescence and in vitro self-renewal capacity. These data highlight choline as a new regulator of HSC maintenance with a conserved function in human and mouse.

## Discussion

Advancing our understanding of the metabolic requirements of human healthy HSCs is critical for the optimization of HSC-based therapies and improving patient outcomes. In this Resource, we provide an integrated metabolic, lipidomic and transcriptomic profile of human BM HSPCs and investigate how their metabolic landscape is changed upon healthy differentiation, physiological ageing and leukaemia.

We first show that human HSPCs have a metabolic profile distinct from that of their downstream progenitors. In particular, we find that human HSPCs, in line with their quiescence state, display lower levels of metabolites that are part of key metabolic pathways such as the TCA cycle and nucleotide and fatty acid metabolism, similar to features previously described in mouse HSCs^13,14,45^. Upon ageing, we observe that human HSPCs suffer metabolic alterations such as increased urea cycle metabolites and reduced levels of NAD and taurine. We also detect fewer acyl-carnitines in aged HSPCs, which is particularly interesting as it has been shown that carnitine supplementation can restore telomere shortening, a hallmark of cellular ageing in HSCs^46^.

In the metabolic profile of AML HSPCs, we observe significant heterogeneity across mutations and metabolic patterns. For example, samples with TET2 mutations are found exclusively in the amino acid high-abundance cluster, indicating a potential relationship between genotype and metabolite levels. These preliminary observations highlight the need for a larger cohort to thoroughly investigate the influence of AML genotypes on metabolite profiles, as understanding these relationships could pave the way for personalized therapeutic strategies tailored to specific genetic backgrounds in AML.

We also find that HSPCs have a distinct lipidomic profile in comparison with their downstream progenitors, which is further altered upon ageing. For instance, there is a higher abundance of long-chain SM in HSPCs, suggesting that they display lower membrane fluidity compared with more differentiated progenitors, as lipids with long and saturated fatty acids make membranes thicker and less fluid owing to the tight packing of their hydrophobic tails and stronger lipid–lipid interactions^47,48^. Meanwhile, the majority of polyunsaturated PC and PE were less abundant in HSPCs compared with progenitors, further supporting the hypothesis that HSPCs display lower membrane fluidity^49–51^. Polyunsaturated lipids are more inclined to lipid peroxidation, a process generating free oxidative radicals. The lower abundance of lipids with polyunsaturated fatty acids in HSPCs might represent a protective mechanism, rendering them more resistant to oxidative damages. Of note, aged HSPCs also showed lower levels of PC, driven by a reduction of the polyunsaturated species, suggesting that they have increased membrane fluidity compared with their young cell counterparts.

Our study highlights choline as a promising new regulator of human HSPCs, as its levels decrease upon differentiation and ageing. In line with this, choline-derived lipids are also affected upon differentiation and ageing. Indeed, when we treated HSPCs with stable isotope-labelled choline, we found it utilized primarily for lipid production, and RNA-seq analyses further supported that it mainly affects lipid metabolism. Choline treatment led to the maintenance of an HSC-like molecular profile, enhanced quiescence and improved in vitro self-renewal capacity of human HSPCs. It is therefore tempting to speculate that the intracellular choline levels regulate lipid composition, resulting in the accumulation of SM. SM, as a key component of lipid rafts, alongside cholesterol, has been characterized as a crucial regulator of mouse HSC function^20,21,52,53^. Furthermore, sphingolipid metabolism has been implicated in human cord blood HSC function through its modulation of proteostatic quality control systems^24^. While our data reveal new avenues for understanding the role of choline in HSC regulation, further studies will be needed to uncover its precise mechanisms and define its in vivo role in HSC regulation.

Furthermore, we observed reduced choline levels in HSPCs from patients with AML, indicating disruptions in lipid-related pathways raising the question whether lipid composition is also relevant in leukaemia. Indeed, disruption of lipid homeostasis through nicotinamide phosphoribosyltransferase inhibition has been shown to selectively induce apoptosis in leukaemic stem cells^54^. Alterations in polyunsaturated fatty acid metabolism can also increase cell susceptibility to ferroptosis, a form of iron-dependent cell death driven by lipid peroxidation. In line with this, AML cells and HSCs under other pathological conditions such as BM failure have been shown to be particularly sensitive to ferroptosis induction^19,55^. Moreover, components of lipid metabolism, such as sphingolipids in lipid rafts and the fatty acid composition of membrane phospholipids, have been implicated in receptor clustering and oncogenic signalling in cancer^48,56–58^. It would be interesting to determine whether such mechanisms also play a role in leukaemic HSCs.

Overall, advances in low-input omics techniques, developed by us and others, have paved the way for profiling rare cell populations, ranging from 10,000 cells to the single-cell level^13–16,23,59^. Valuable studies on human HSPCs, particularly from cord blood and mobilized peripheral blood^19,24^, have demonstrated the importance of lipidomic and metabolomic analyses in understanding stem cell biology. In this study, to assess the HSPC lipid composition upon differentiation, ageing and leukaemia, we optimized a low-input untargeted lipidomics workflow, combining FACS with LC–MS analysis, which can now be applied to other rare cell populations. While this approach has provided a broad overview into lipid profiles, enhancing our lipidomics methodology could yield even greater resolution in future studies. Addressing challenges related to low-input material and incorporating comprehensive isomer detection will further enrich our understanding of lipid metabolism in HSCs.

Taken together, our study provides a comprehensive metabolic profile of human BM-derived HSPCs upon differentiation, ageing and AML. In the future, these metabolic features can provide opportunities for the development of new therapeutic strategies that target the maintenance of human HSCs.

## Methods

### Human BM specimens

Our research complies with all relevant ethical regulations. Human BM specimens were obtained from patients who provided written consent to participate in this study under one of the following approved ethical protocols: Ethical Committee University Hospital Cologne/Sign: 22-1095; Ethical Committee University Hospital Freiburg/Sign: 22-1047 and 20-1253; Ethical Committee Frankfurt University Hospital SHN-07-2015; Johns Hopkins Institutional Review Board; Ethical Committee of the Medical Faculty of Heidelberg (S-169/2017 and S-648/2021). Pseudonymized left-over samples from healthy BM donors (rinseback from BM filters) were used with written informed consent in accordance with vote #329/10 (Ethics Committee of Goethe University Medical Center). Additional details are provided in Supplementary Table 1. Freshly isolated BM samples were collected in EDTA, and BM mononuclear cells were purified using Lymphoprep (STEMCELL Technologies, #07851). Subsequently, samples were frozen using 10% dimethyl sulfoxide in foetal calf seru at −80 °C and stored long-term in liquid nitrogen. Commercially available CD34-enriched BM samples were purchased from StemCell Technologies (human BM CD34^+^; #70002.4). In brief, primary human CD34^+^ cells were isolated from BM mononuclear cells using positive immunomagnetic separation techniques and cryopreserved in serum-free medium. Cells were collected using institutional review board-approved consent forms and protocols. Heparin is added during collection as an anticoagulant.

### Human sample preparation

Human frozen BM samples were thawed at 37 °C and then transferred to 10 ml of thawing medium (IMDM; 10% foetal calf serum, 1 mM EDTA and 1:1,000 DNase). They were subsequently resuspended in StemSpan medium (StemSpan SFEM II #09605) and allowed to recover in the incubator for 20 min. After this, the cells were resuspended in 500 μl of antibody mix. Cells were stained using the following antibodies (all obtained from BioLegend): CD34-FITC (cat. no. 343504, 1:100) or CD34-AF488 (cat. no. 343518, 1:100), CD38-PE/Cy7 (cat. no. 303516, 1:100) or CD38-AF700 (cat. no. 343518, 1:100), and Lineage-APC (CD3, CD14, CD16, CD19, CD20, CD56; cat. no. 348803, 1:100) and incubated at 4 °C for 45 min. After the incubation, the cells were washed with 0.9% NaCl and incubated with Zombie-Aqua (cat. no. 423101, 1:1,000) for 10 min at room temperature. Finally, the cells were washed and resuspended in 0.9% NaCl and filtered for FACS.

### Isolation of murine BM cells

C57BL/6J mice were euthanized by CO_2_ inhalation followed by cervical dislocation according to guidelines and animal protocols approved by the German authorities. Using forceps and scissors, legs and spines were dissected. To isolate femurs, tibiae, iliae and vertebrae, connective tissue was removed with a scalpel. The isolated bones were gently crushed twice with 5 ml of ice-cold PBS (Sigma, D8537) using a mortar and pestle, and the cell suspension was then filtered through a 40-μm sterile filter (Corning, 352340) into a 50-ml Falcon tube. Cells were subsequently erylysed with ACK Lysis Buffer (Lonza, 10-548E) and washed with ice-cold PBS. Lineage depletion was performed using the Dynabeads Untouched Mouse CD4 Cells Kit (Invitrogen, 11415D). Cells were then stained with the following antibodies (all obtained from BioLegend unless stated otherwise): Lineage-BV650 (Gr1 (cat. no. 108442, 1:1,000), CD11b (cat. no. 101259, 1:1,000), B220 (cat. no. 103241, 1:500), Ter119 (cat. no. 116235, 1:500), CD4 (cat. no. 563232, 1:1,000), CD8a (cat. no. 100722, 1:1,000), cKit-BV711 (cat. no. 105835, 1:1,000), Sca1-APC-Cy7 (cat. no. 108126, 1:500), CD150-PeDazzle (cat. no. 115936, 1:800), CD48-PE/Cy7 (cat. no. 103424, 1:1,000), CD16/32-APC (cat. no. 101326, 1:1,000) and CD34-FITC (BD Biosciences, cat. no. 553733, 1:50) 45 min at 4 °C. After staining, cells were washed with 0.9% NaCl and filtered for FACS.

### In vitro culture of HSPCs

Human HSPCs were cultured in StemSpan medium (StemSpan SFEM II #09605) containing Supplement (StemSpan CD34^+^ Expansion Supplement (10×) #02691), GlutaMax 1× and low-density lipoprotein at 10 μg ml^−1^ in atmospheric conditions.

Mouse HSCs were cultured in Complete Stem Cell Medium (StemPro-34 SFM, Life Technologies, supplemented with 50 ng ml^−1^ stem cell factor, 25 ng ml^−1^ thrombopoietin, 30 ng ml^−1^ Flt3-Ligand (all from Preprotech), 100 µg ml^−1^ penicillin–streptomycin and 2 mM l-glutamine (both from Gibco)) in atmospheric conditions.

### In vitro CFU assays

For human HSPCs, 1,000 HSCs (Lin^−^ CD34^+^ CD38^−^) were treated for 48 h with 10 mM choline chloride (aq) (Sigma, C7017) in 100 μl StemSpan medium. Cells were then plated in MethoCult H4435 (STEMCELL Technologies), and colonies were counted after 8 days. A total of 10,000–20,000 cells were serially replated and counted after 8 days.

For mouse HSCs, 300 HSCs (Lin^−^ cKit^+^ Sca1^+^ CD150^+^ CD48^−^ CD34^−^) were treated for 48 h with 5 mM choline chloride (aq) (Sigma, C7017) in 100 μl Stem Pro complete. Cells were then plated in MethoCult M3434 (STEMCELL Technologies), and colonies were counted after 5 days. A total of 10,000 cells were serially replated and counted after 5–7 days and up to three platings.

### Annexin V-staining

Cells were stained with Annexin V following standard procedures (ThermoFisher Scientific, #88-8007-74). In brief, cells were washed in phosphate-buffered saline (PBS) and 1× binding buffer before resuspension at 1–5 × 10^6^ cells ml^−1^. Annexin V-APC (2 μl) was added to 50 μl of the cell suspension and incubated for 10–15 min at 4 °C. After washing, cells were resuspended in 1× binding buffer, stained with 4′,6-diamidino-2-phenylindole (Sigma, #D9542, 1:500) and analysed by flow cytometry within 4 h.

### In vitro single-cell division assays

Human HSPCs (Lin^−^ CD34^+^ CD38^−^) and mouse HSCs (Lin^−^ cKit^+^ Sca1^+^ CD150^+^ CD48^−^ CD34^−^) were single sorted into 72-well Terasaki plates (Greiner Bio-One) in StemSpan complete and Stem Pro complete, respectively. After a 48-h incubation period, each well was manually examined to determine the number of cell divisions: one cell indicated no division, two cells indicated one division, and more than two cells indicated more than one division. Flow cytometry data from functional analyses were processed using FlowJo software (BD Biosciences), and statistical analyses were performed using GraphPad Prism.

### Targeted metabolomics

For low-input targeted metabolomics of polar metabolites, 1,000–5,000 cells were FACS sorted and processed as previously described^16,60^ with minor modifications. In brief, cells were sorted directly in 25 μl of extraction buffer containing ^13^C-labelled yeast extract as internal standard (ISOtopic solutions, ISO-1) in acetonitrile (LC–MS grade). FACS was conducted using a 70-μm nozzle and water with 2 g l^−1^ NaCl as sheath fluid. With these conditions, each FACS droplet has a volume of 1 nl; consequently, 5,000 cells come with a total of 5 µl of sheath fluid. To ensure comparability, process blank (NC) samples were generated, which serve as controls to monitor background impurities introduced during the workflow. To this end, 5 µl of residual sheath fluid from the sheath fluid tank was mixed with 25 µl of extraction buffer. All samples were centrifuged (10 min at 1,600*g*), and 25 µl of clear supernatant was transferred to a 96-well plate for LC–MS analysis. Targeted metabolite quantification by LC–MS was carried out using an Agilent 1290 Infinity II UHPLC in line with an Agilent 6495 QQQ-MS operating in ‘Dynamic MRM’ mode. MRM settings were optimized separately for all compounds using pure standards (see Supplementary Table 9 for compound-specific MS parameters). The cycle time was 1,000 ms, resulting in dwell times between 2.7 ms and 165 ms. LC separation was performed on a Waters Atlantis Premier BEH ZHILIC column (100 × 2.1 mm, 1.7 µm particles), buffer A was 20 mM ammonium carbonate and 5 µM medronic acid in MilliQ H_2_O, buffer B was 90:10 acetonitrile:buffer A, and the solvent gradient was from 95% to 55% buffer B over 18 min. The flow rate was 150 µl min^−1^, column temperature was 40 °C, autosampler temperature was 5 °C and injection volume was 20 µl. Data processing was performed using the R package automRm^60^.

Metabolites were identified on the basis of retention times and at least two known fragments, one of which was used as quantifier to determine the area under the curve (AUC) of the chromatographic peak as a measure for metabolite abundance. Because the same number of cells were extracted for each sample, no further data scaling or normalization was performed. Missing values were handled per individual experiment before merging the data. In brief, if a metabolite was detected in at least one sample, missing values for that metabolite were set to zero. When a metabolite was entirely missing across all samples within an experiment, the missing values were retained. Data analysis was performed on a linear scale with the exception of fold changes, which were represented on a logarithmic scale for visualization purposes. For comparisons between human and mouse HSPCs and progenitors, as well as between choline-treated and control HSPCs, paired samples were sorted from the same biological individuals. In these datasets, metabolites exhibiting higher abundance in any condition than the mean of the blank samples (negative control, NC) per experiment were selected for further analysis. Moreover, metabolites that were significantly more abundant in all biological and technical replicates than in NC after merging experiments were considered detected above background levels in each dataset (*P* < 0.1). To this end, a Shapiro–Wilk test per metabolite was applied to test for normality, and significant abundance was assessed by unpaired Student’s *t*-test or unpaired Wilcoxon test in the case of normal or non-normal distribution, respectively. The mean of metabolite abundance in technical replicates was calculated to obtain a single value per biological replicate. Finally, metabolites with a higher average abundance than 100 in any condition and detected in three or more biological replicates per condition were considered detected per dataset. Metabolite abbreviations used along the manuscript are indicated in the corresponding supplementary tables.

To assess metabolite abundance differences between conditions, Student’s *t*-test or Wilcoxon test were used depending on normality as detailed above. This test was paired only in HSPC versus progenitor or choline-treated versus control HSC comparisons as they originated from the same biological individuals. Adaptive multiple hypothesis correction was assessed by the CAMT package, using the metabolite average intensity as a covariate to control for false discovery rate^61^. Metabolites with *P*-adjusted <0.05 were considered significantly different. In the analysis of AML versus healthy individuals, some samples were processed immediately after sorting, while others were processed and then frozen. To account for potential differences due to processing, metabolite values were normalized to their average abundance per freezing condition before conducting significance testing. Metabolite abundances were visualized using bar plots. Individual biological replicate differences were visualized with heat maps showing the log_2_fold change (FC) value between each paired HSPC–progenitor and choline-treated–control HSPC biological sample. In the other datasets, log_2_FC between each individual biological replicate of the condition of interest and the mean abundance of the other condition were represented. When resulting log_2_FC values are Infinity or −Infinity because the absolute abundance in one of the conditions is 0, log_2_FC is set to the maximum or minimum value in the dataset, respectively. Volcano plots of the differentially abundant metabolites were represented with EnhancedVolcano^62^. Dimensional reduction of the samples based on the significantly variable metabolites was performed through PCA. Correlation of metabolite abundance and patient age was represented using geom_smooth(method = lm) and stat_cor(method = “pearson”).

### Lipidomics

A total of 5,000 cells were FACS sorted into vials with integrated glass microinsert (Fisherbrand 11864910) containing 15 μl of lipid extraction buffer (2:1 2-propanol:acetonitrile). A 70-μm nozzle and water with 2 g l^−1^ NaCl as sheath fluid were utilized during FACS to achieve a final extracted volume of 20 μl with a composition of 2:1:1 2-propanol:acetonitrile:water. For absolute quantification, Splash standard (cat. no. 330707-1EA, diluted 1:2,000 in the extraction buffer) was added where absolute concentrations are indicated. Immediately after the sort, sample vials were flicked and tapped on a table surface to ensure mixing of the sample and location of the sample in the bottom of the vial. We opted for this one-phase protocol for lipid extraction to circumvent possible loss of material that could occur in a multiphase extraction. Using SPLASH lipid standards, as a control, we observed virtually identical signal intensities between NCs and cells, suggesting consistent recovery.

Non-targeted measurement of lipids by LC–MS was carried out as described previously by Edwards-Hicks and colleagues^34^ using an Agilent 1290 Infinity II UHPLC inline with a Bruker Impact II QTOF-MS equipped with an Bruker ionBooster heated electrospray ionization source. LC separation was on a Zorbax Eclipse plus C18 column (100 × 2 mm, 1.8-µm particles) using a solvent gradient of 70% buffer A (10 mM ammonium formiate in 60:40 acetonitrile:water) to 97% buffer B (10 mM ammonium formiate in 90:10 2-propanol:acetonitrile). The flow rate was 400 µl min^−1^, autosampler temperature was 5 °C and injection volume was 17 µl. The mass spectrometer was operated in positive ion mode with a scan range from 50 to 1,600 *m*/*z*. MS2 events were recorded by data-dependent acquisition. Mass calibration was performed at the beginning of each sample run. A detailed list of data acquisition parameters including LC gradient, ion source parameters, ion optics parameters and parameters for selection and exclusion of features for MS2 spectra acquisition can be found in Supplementary Table 2. Data processing including feature detection, feature deconvolution and annotation of lipids was performed using MetaboScape (version 2023b). Peaks were detected using the build-in T-ReX 3D algorithm, and peak areas were used as a measure for abundance of lipids. The most important parameters for feature detection and feature deconvolution are summarized in the table below. A more detailed list of parameters including parameters for feature extraction, recalibration of the *m*/*z* axis and feature annotation can be found in Supplementary Table 2. Because the same number of cells were extracted for each sample, no further data scaling or normalization was performed. Data analyses were performed on a linear scale with the exception of fold changes, which were represented on a logarithmic scale.

Features with a *m*/*z* mass below 300 and retention time below 2 min were excluded from the analysis. Total acyl-chain length and unsaturations are shown in all annotated lipids along the manuscript. In addition, lipid isomers with annotated single acyl chains are depicted in their detailed form in Supplementary Table 9 when accurately detected. When the order of every acyl chain in the lipid is known or unknown, they are separated by ‘/’ and ‘_’ symbols, respectively. In each individual experiment, lipid isomers with the same formula and annotated name were merged, adding their abundance per replicate. Then, annotated lipids sharing both name and formula across experiments were considered the same detected lipid. For all analyses, only samples from the two conditions of interest that were processed within a comparable time frame were included to ensure consistency. The methods for missing data handling, compound selection, analysis and visualization of detected lipids were identical to the previously mentioned metabolomics pipeline. For calculation of absolute lipid concentration in samples where Splash was added, concentration and signal intensity of each lipid class standard were used, accounting for type 1 isotope correction by using the Rdisop package^63^. Heat maps (visualized as a lipid membrane, ‘Lipheat’) representing the ordered log_2_FC between the average lipid abundance per condition were generated.

Detected lipids were grouped in main lipid classes. Dot plots depicting the total number of carbons (length) and double bounds in the acyl chains of detected lipids categorized by class were generated. SMs were subdivided into short-chain (<36 carbons) and long-chain (≥36 carbons) subclasses. All other lipid classes were categorized on the basis of the saturation levels of their acyl chains: saturated (0 double bonds), MUFA/monounsaturated (1 double bond) and PUFA/polyunsaturated (≥2 double bonds). To assess differences between conditions in lipid classes or subclasses, the sum of the average lipid AUC per condition was calculated, as their similar head confers them similar ionization and flight characteristics. To assess significance between conditions, Student’s *t*-test or Wilcoxon test were used depending on normality, and adaptive multiple hypothesis correction was assessed by the CAMT package^61^, as previously detailed in ‘Targeted metabolomics’ section.

### Stable isotope labelling

A total of 10,000 human HSPCs (Lin^−^ CD34^+^ CD38^−^) were sorted and treated with ^13^C-labelled choline chloride (Choline Chloride-13C-3, HY-B1337S4, MedChemExpress) for 72 h. After treatment, cells were resorted and prepared for subsequent analyses: 1,000 cells were resorted into 80% acetonitrile (final concentration, for targeted metabolomics), and 5,000 cells were resorted in 2:1:1 mixture of 2-propanol:acetonitrile:water (final concentration, for lipidomics). Chromatographic separation and mass spectrometry parameters were identical to metabolomics and lipidomics analyses, respectively, with the sole difference that data were recorded in MS1 mode only with a scan frequency of 2 Hz to maximize signal intensity and dynamic range. Raw data were converted to mzML format using ms-convert. Subsequent data analysis, including correction for natural isotope distribution, was performed in R as described previously^64,65^ (Supplementary Data 2). In brief, metabolite and lipid peaks were identified on the basis of exact mass and known retention times of standard metabolites and lipids. To confirm the identity of lipids, a quality control sample was included in the same batch and analysed including MS2 by data-dependent acquisition. Only those lipids were used for stable isotope label tracing, for which an MS2 confirmed peak was detected within 15 s and 1 mDa tolerance (Supplementary Table 8). Next, chromatographic peaks for all relevant isotopologues were summed to determine the start and end of the integration range in the retention time (RT) dimension. Peak areas were determined with identical peak borders for all isotopologues. Subsequently, correction for natural abundance of isotopologues was calculated using code from the R package accucor by least-squares fitting a linear combination of theoretical distributions. Normalized mass distribution vectors were used to calculate the ratio of labelled (M + 3 for choline, phosphocholine, cytidine diphosphate-choline (CDP-choline), betaine and lipids; M + 1 for methionine and SAM) to non-labelled fraction (M + 0) for each treated sample, and the averages of these ratios were represented in pie charts.

### Population RNA-seq

#### Nucleic acid extraction protocol

Cells were sorted intoRNA lysis buffer (Arcturus PicoPure RNA Isolation Kit (Applied Biosystems) and then stored at −80 °C until further use. RNA isolation was conducted using the Arcturus PicoPure RNA Isolation Kit (Applied Biosystems) following the manufacturer’s guidelines. DNase treatment was carried out using the RNase-Free DNase Set (Qiagen). The resulting total RNA was utilized for generating cDNA libraries.

#### Nucleic acid library construction protocol and sequencing

cDNA libraries were generated using SMARTseq v4 (Takara Bio). Amplification cycles were adjusted accordingly to RNA input amount. For HSPCs, 13 cycles of amplification were performed. To produce uniquely and dually barcoded sequencing libraries from the cDNA libraries, the NEBNext Ultra II FS DNA library kit was utilized. This involved fragmenting of 3.5–5 ng of the cDNA library for 22.5 min, followed by adaptor ligation and library amplification using cycle numbers determined by the amount of input material. Libraries underwent sequencing on the Illumina NovaSeq platform with 100-bp paired-end sequencing.

#### Population RNA-seq analysis method—low-level processing

Raw FASTQ files were aligned against the hg38 reference genome using the mRNA-seq tool of the pipeline snakePipes v.2.5.2 (ref. ^66^). Alignment was performed using STAR (STAR_2.7.4a), with the Alignment mode utilized for mapping the sequenced reads^67^. Subsequently, gene counts were quantified using featureCounts^68^. Data quality assessment was conducted using Deeptools QC v3.3.2 (ref. ^69^). For further analysis, genes with an average expression exceeding 100 counts in at least one condition were specifically selected. Differential expression analysis was carried out using DESeq2 (ref. ^70^), with results considered statistically significant at a false discovery rate of 0.05.

#### Population RNA-seq analysis method—downstream analysis

For evaluation of expression of Gene Ontology (GO) biological processes, metabolic pathways^14^ and quiescent long term (LT)-HSC versus short term (ST)-HSC signature^71^ in the pairwise comparisons, GSEA was performed with the fgseaMultilevel(nPermSimple = 10,000) function from the fgsea package^72^, selecting significant pathways below a false discovery rate equal to 0.1. The resulting normalized enrichment score (NES) and *P*-adjusted value of selected signatures were represented with ggplot2 (ref. ^73^). Volcano plots were represented with EnhancedVolcano^62^. Expression values of differentially expressed Kyoto Encyclopedia of Genes and Genomes (KEGG) enzymes (log_2_FC threshold of 1, *P*-adjusted <0.05) per pairwise comparison were variance stabilizing transformed, relativized across samples and represented in a heat map. TF and pathway activity scores were inferred by decoupleR^29^ as per developers vignettes based on the stat measurement from DESeq2 per pairwise comparison. For TF activity estimation, a univariate linear model was applied to calculate the linear correlation between gene expression and TF–gene interaction weights, based on CollecTRI, a curated database of TFs and their targets^74^. In the case of pathway activity, multivariate linear model and PROGENy pathways–target gene interactions were used. Additionally, i-cisTarget was used to predict the presence of TF regulatory motifs in co-expressed genes upon choline treatment (log_2_FC >0.25, *P*-adjusted <0.05)^75^.

### Integration of omics datasets

To examine correspondence between metabolomic and transcriptional profiles on a per-sample basis, we performed Pearson correlation between metabolite intensity and enzyme expression in each KEGG pathway. To integrate perturbations across different omics datasets at a metabolic pathway level in an unbiased manner, we custom-generated metabolograms (metabolome–transcriptome plots) following the method outlined by Hakimi and colleagues^32^. Each pathway’s metabolites and the individual enzymatic genes detected in RNA-seq from the respective KEGG pathway are depicted as slices in the periphery. The colour scale represents the log_2_FC of differential metabolite abundance and enzyme expression. The individual *P*-adjusted value of metabolites log_2_FC are calculated as previously described in ‘Targeted metabolomics’ and ‘Lipidomics’ sections, while RNA-seq significance is determined by DESeq2 (*P < 0.05, **P < 0.01, ***P < 0.001). In addition, the average log_2_FC values of enzymes and metabolites are displayed in the centre, and a Wilcoxon test was used to calculate the significance of the change based on the individual measurements. Only pathways with at least five detected enzymes in RNA-seq and one measured metabolite are presented. To establish mechanistic hypotheses of regulation across our omics datasets, we utilized the computational tool COSMOS based on the developers vignettes^76^, with the parameter maximum_network_depth = 5. Based on the log_2_FC values of our metabolomics and transcriptomics datasets, the estimated TF activity by decoupleR and their generated prior knowledge network, COSMOS finds connected deregulation events in the data solving an optimization problem with the tool CARNIVAL. Gene nodes that showed an opposite trend in activity and significantly different expression were excluded from the final network.

### Statistics and reproducibility

No statistical methods were used to predetermine sample sizes. No data were excluded from the analyses. Data distribution was assumed to be normal, but this was formally tested only when stated. The experiments were not randomized. The investigators were not blinded to allocation during experiments and outcome assessment.

### Reporting summary

Further information on research design is available in the Nature Portfolio Reporting Summary linked to this article.

## Online content

Any methods, additional references, Nature Portfolio reporting summaries, source data, extended data, supplementary information, acknowledgements, peer review information; details of author contributions and competing interests; and statements of data and code availability are available at 10.1038/s41556-025-01709-7.

## Supplementary information

Reporting Summary

Supplementary Table 1 Demographic and clinical characteristics of patients, analysis classification and sample identifiers.

Supplementary Table 2 Lipidomics acquisition and processing parameters.

Supplementary Table 3 Quality control metabolomics and lipidomics data of human HSPCs and progenitors, related to Figs. 1 and 2.

Supplementary Table 4 Metabolomics, lipidomics and RNA-seq data of human HSPCs and progenitors, related to Figs. 1 and 2.

Supplementary Table 5 Metabolomics and lipidomics data of mouse HSCs and progenitors, related to Fig. 3.

Supplementary Table 6 Metabolomics, lipidomics and RNA-seq data of human aged and young HSPCs, related to Fig. 4.

Supplementary Table 7 Metabolomics and RNA-seq data of human AML and healthy HSPCs, related to Fig. 5.

Supplementary Table 8 Metabolomics, lipidomics, stable isotope labelling and RNA-seq data of choline-treated and control HSCs/HSPCs, related to Fig. 6.

Supplementary Table 9 Targeted metabolomics parameters and lipidomics raw annotated data per experiment.

Supplementary Data 1 Lipidomics method reporting checklist.

Supplementary Data 2 R script for raw data processing for ^13^C-labelling experiments.

## Source data

Source Data Fig. 6 Source data.

Source Data Extended Data Fig. 9 Source data.

## Data Availability

An interactive application visualizing all genes, metabolites and lipids detected in human HSPCs, along with their differential abundance across differentiation, ageing and leukaemia, is available at https://cabezas-lab.shinyapps.io/HumanMetabolomics/. The raw transcriptomics data were deposited in ArrayExpress and are available under the accession numbers E-MTAB-13862 and E-MTAB-13863. The raw metabolomics and lipidomics data were deposited in MassIVE under the accession number MSV000097228. Source data are provided with this paper.

## Code availability

Further information on the code used in this study is provided in the Methods and Supplementary Data 2. Any other requests should be addressed to J.B. and N.C.-W.
