# Supplementary material for: Differentiation, ageing and leukaemia alter the metabolic profile of human bone marrow haematopoietic stem and progenitor cells
Source: Nat Cell Biol. 2025 Jul 15;27(8):1367–80. doi: 10.1038/s41556-025-01709-7 (PMC12339397; doi:10.1038/s41556-025-01709-7)
Supplement: Supplementary file 1 — Reporting Summary [file 41556_2025_1709_MOESM1_ESM.pdf]

nature portfolio

Corresponding author(s): Joerg Buescher, Nina Cabezas-Wallscheid

Last updated by author(s): Apr 16, 2025

Reporting Summary

Nature Portfolio wishes to improve the reproducibility of the work that we publish. This form provides structure for consistency and transparency in reporting. For further information on Nature Portfolio policies, see our Editorial Policies and the Editorial Policy Checklist.

Statistics

For all statistical analyses, confirm that the following items are present in the figure legend, table legend, main text, or Methods section.

n/a	Confirmed	
☐	☒	The exact sample size (*n*) for each experimental group/condition, given as a discrete number and unit of measurement
☐	☒	A statement on whether measurements were taken from distinct samples or whether the same sample was measured repeatedly
☐	☒	The statistical test(s) used AND whether they are one- or two-sided<br>*Only common tests should be described solely by name; describe more complex techniques in the Methods section.*
☒	☐	A description of all covariates tested
☐	☒	A description of any assumptions or corrections, such as tests of normality and adjustment for multiple comparisons
☐	☒	A full description of the statistical parameters including central tendency (e.g. means) or other basic estimates (e.g. regression coefficient) AND variation (e.g. standard deviation) or associated estimates of uncertainty (e.g. confidence intervals)
☐	☒	For null hypothesis testing, the test statistic (e.g. *F*, *t*, *r*) with confidence intervals, effect sizes, degrees of freedom and *P* value noted<br>*Give P values as exact values whenever suitable.*
☒	☐	For Bayesian analysis, information on the choice of priors and Markov chain Monte Carlo settings
☒	☐	For hierarchical and complex designs, identification of the appropriate level for tests and full reporting of outcomes
☒	☐	Estimates of effect sizes (e.g. Cohen's *d*, Pearson's *r*), indicating how they were calculated

Our web collection on statistics for biologists contains articles on many of the points above.

Software and code

Policy information about availability of computer code

Data collection	snakePipes v.2.5.2 bioinformatics pipeline<br>EnhancedVolcano v.1.24.0, fgsea v.1.32.4, ggplot2 v.3.5.2, Rdisop v.1.66.0, decoupleR v.2.12.0, COSMOS v.1.14.0, CAMT v.1.1 and automRm R packages<br>FlowJo v10.10 and Graphpad Prism v.10.4.2 softwares
Data analysis	Targeted Metabolomics analysis<br>For comparisons between human and mouse HSCs and progenitors, as well as between choline-treated and control HSCs, paired samples were sorted from the same biological individuals. In these datasets, metabolites exhibiting higher abundance in any condition than the mean of the blank samples (Negative Control; NC) per experiment were selected for further analysis. Moreover, metabolites that were significantly more abundant in all biological/technical replicates than in NC were considered detected above background levels in each dataset. Mean of metabolite abundance in technical replicates was calculated to obtain a single value per biological replicate. Finally, metabolites with a higher average abundance than 100 in any condition and detected in three or more biological replicates per condition were considered detected per dataset. To assess metabolite abundance differences between conditions, Student's t test or Wilcoxon test were used depending on normality. Adaptive multiple hypothesis correction using the metabolite average intensity as a covariate was assessed. Metabolites with p-adjusted < 0.05 were considered significantly different. Individual biological replicate differences were visualized with heatmaps showing the log2FC value per comparison. Volcano plots of the differentially abundant metabolites were represented with EnhancedVolcano. Dimensional reduction of the samples based on their metabolic landscape was performed through Principal Component Analysis (PCA).<br><br>Lipidomics<br>The methods for selection, analysis and visualization of detected lipids were identical to the previously mentioned metabolomics pipeline. Additionally, heatmaps representing the ordered log2FC between the average lipid abundance per condition were generated. Dotplots

depicting the total number of carbons (length) and double bounds in the acyl-chains of detected lipids categorized by class were generated. To assess differences between conditions in lipid classes or subclasses, the sum of the average lipid abundances per condition was calculated, as their similar head confers them similar ionization and flight characteristics. To assess significance between conditions, Student's t test or Wilcoxon test were used depending on normality.

Population RNA analysis: low-level processing
Raw FASTQ files were aligned against the hg38 reference genome using the mRNA-seq tool of the pipeline snakePipes. For further analysis, genes with an average expression exceeding 100 counts in at least one condition were specifically selected. Differential expression analysis was carried out using DESeq2, with results considered statistically significant at a false discovery rate (FDR) of 0.05.

Population RNA analysis: downstream analysis
For evaluation of expression of Gene Ontology (GO) biological processes and metabolic pathways in the pairwise comparisons, GSEA was performed with the fgsea package. Transcription factor and pathway activity scores were inferred by decoupleR as per developers vignettes. Additionally, i-cisTarget was used to predict the presence of TF regulatory motifs in co-expressed genes.

Integration of omics datasets
In order to integrate perturbations across different omics datasets at a metabolic pathway level in an unbiased manner, we custom-generated metabolograms (Metabolome-Transcriptome plots). To establish mechanistic hypotheses of regulation across our omics datasets, we utilized the computational tool COSMOS based on the developers vignettes.

For manuscripts utilizing custom algorithms or software that are central to the research but not yet described in published literature, software must be made available to editors and reviewers. We strongly encourage code deposition in a community repository (e.g. GitHub). See the Nature Portfolio guidelines for submitting code & software for further information.

Data

Policy information about availability of data

All manuscripts must include a data availability statement. This statement should provide the following information, where applicable:

- Accession codes, unique identifiers, or web links for publicly available datasets
- A description of any restrictions on data availability
- For clinical datasets or third party data, please ensure that the statement adheres to our policy

The raw transcriptomics data were deposited in ArrayExpress and are available under the accession numbers E-MTAB-13862 and E-MTAB-13863. The raw metabolomics/lipidomics data were deposited in MassIVE under the accession number MSV000097228. An app depicting all genes, metabolites and lipids detected in human HSPCs and representing their differential abundance in differentiation, aging and leukemia, is publicly available in the web link https://cabezas-lab.shinyapps.io/HumanMetabolomics/

Research involving human participants, their data, or biological material

Policy information about studies with human participants or human data. See also policy information about sex, gender (identity/presentation), and sexual orientation and race, ethnicity and racism.

Reporting on sex and gender	The study incorporates samples from both male and female sexes. There are no exclusion criteria based on sex. Gender information has not been collected.
Reporting on race, ethnicity, or other socially relevant groupings	Race or ethnicity data has not been collected.
Population characteristics	Population characteristics of the human research participants are collected in Supplementary Table 1.
Recruitment	BM biopsies were obtained from consenting donors or hip replacement surgeries, excluding individuals with infectious diseases. Non-AML patients were included only if free from hematological disorders. Hip replacement surgery patients were included if bone marrow access was necessary (e.g., arthroplasty). Human bone marrow specimens were also purchased from StemCell Technologies (human BM CD34+; #70002.4).
Ethics oversight	Human bone marrow specimens were either purchased from StemCell Technologies (human BM CD34+; #70002.4) or obtained from patients who provided written consent to participate in this study under one of the following approved ethical protocols: Ethical Committee University Hospital Cologne/Sign: 22-1095; Ethical Committee University Hospital Freiburg/Sign: 22-1047 and 20-1253; Ethical Committee Frankfurt University Hospital SHN-07-2015; Johns Hopkins Institutional Review Board and vote #329/10 (Ethics Committee of Goethe University Medical Center). Additional details are provided in Supplementary Table 1.

Note that full information on the approval of the study protocol must also be provided in the manuscript.

Field-specific reporting

Please select the one below that is the best fit for your research. If you are not sure, read the appropriate sections before making your selection.

☒ Life sciences ☐ Behavioural & social sciences ☐ Ecological, evolutionary & environmental sciences

For a reference copy of the document with all sections, see nature.com/documents/nr-reporting-summary-flat.pdf

Life sciences study design

All studies must disclose on these points even when the disclosure is negative.

Sample size	Sample size was determined based on extensive experience with similar experiments in our laboratory (Cabezas-Wallcheid et a., 2014 and 2017; Sommerkamp et al., 2020 and 2021; Renders et al., 2021).
Data exclusions	No data were excluded from this study.
Replication	Replication was successful in every attempt. Key and possible experiments were performed at least twice, exact number of independent experiments with several biological replicates are shown in figure legends.
Randomization	All patients samples/mice were analyzed and allocated randomly.
Blinding	No blinding experiments were needed, as values were quantitative comparisons as determined by software and measurement.

Reporting for specific materials, systems and methods

We require information from authors about some types of materials, experimental systems and methods used in many studies. Here, indicate whether each material, system or method listed is relevant to your study. If you are not sure if a list item applies to your research, read the appropriate section before selecting a response.

Materials & experimental systems

n/a	Involved in the study	
☐	☒	Antibodies
☒	☐	Eukaryotic cell lines
☒	☐	Palaeontology and archaeology
☐	☒	Animals and other organisms
☒	☐	Clinical data
☒	☐	Dual use research of concern
☒	☐	Plants

Methods

n/a	Involved in the study	
☒	☐	ChIP-seq
☐	☒	Flow cytometry
☒	☐	MRI-based neuroimaging

Antibodies

Antibodies used

CD11b-BV650 BioLegend 101259; RRID:AB_2566568
Gr1-BV650 BioLegend 108442; RRID:AB_2686974
TER119-BV650 BioLegend 116235; RRID:AB_11204244
B220-BV650 BioLegend 103241; RRID:AB_11204069
CD4-BV650 BioLegend 563232; RRID:AB_2738083
CD8a-BV650 BioLegend 100742; RRID:AB_2563056
cKit-BV711 BioLegend 105835; RRID:AB_2565956
Sca1-APC/Cy7 BioLegend 108126; RRID:AB_10645327
CD150-PE/Dazzle Biolegend 115936 RRID:AB_2565960
CD48-Pe/Cy7 Biolegend 103424 RRID AB_2075049
CD34-FITC BD Biosciences 553733; RRID:AB_395017
hLineage-APC (CD3, CD14, CD16, CD19, CD20, CD56) BioLegend 348803
hCD34-FITC BioLegend 343504; RRID: AB_1731852
hCD34-AF488 BioLegend 343518 RRID:AB_1937203
hCD38-AF700 BD Biosciences 560676; RRID: AB_1727472
hCD38-PeCy7 BioLegend 303516; RRID: AB_1279235

Validation

All flow cytometry were already established and commonly used in our laboratory and have been published multiple times by us and other groups, as described in Cabezas-Wallscheid et al., 2017, Cell; Schoenberger et al,, 2022 CSC; Zhang et al., 2022 NCB.

Animals and other research organisms

Policy information about studies involving animals; ARRIVE guidelines recommended for reporting animal research, and Sex and Gender in Research

Laboratory animals	All mice were bred in-house in the animal facility at the MPI-IE in individually ventilated cages (IVCs). Animal procedures were performed according to German guidelines. Female mice between 8-12 weeks of age were used.
Wild animals	No wild animals were used in this study.

Reporting on sex	Female mice between 8-12 weeks of age were used.
Field-collected samples	No field collected samples were used in this study.
Ethics oversight	All mice were bred in-house in the animal facility at the MPI-IE in individually ventilated cages (IVCs). Animal procedures were performed according to German guidelines.

Note that full information on the approval of the study protocol must also be provided in the manuscript.

Plants

Seed stocks	*Report on the source of all seed stocks or other plant material used. If applicable, state the seed stock centre and catalogue number. If plant specimens were collected from the field, describe the collection location, date and sampling procedures.*
Novel plant genotypes	*Describe the methods by which all novel plant genotypes were produced. This includes those generated by transgenic approaches, gene editing, chemical/radiation-based mutagenesis and hybridization. For transgenic lines, describe the transformation method, the number of independent lines analyzed and the generation upon which experiments were performed. For gene-edited lines, describe the editor used, the endogenous sequence targeted for editing, the targeting guide RNA sequence (if applicable) and how the editor was applied.*
Authentication	*Describe any authentication procedures for each seed stock used or novel genotype generated. Describe any experiments used to assess the effect of a mutation and, where applicable, how potential secondary effects (e.g. second site T-DNA insertions, mosiacism, off-target gene editing) were examined.*

Flow Cytometry

Plots

Confirm that:

☒ The axis labels state the marker and fluorochrome used (e.g. CD4-FITC).

☒ The axis scales are clearly visible. Include numbers along axes only for bottom left plot of group (a 'group' is an analysis of identical markers).

☒ All plots are contour plots with outliers or pseudocolor plots.

☒ A numerical value for number of cells or percentage (with statistics) is provided.

Methodology

Sample preparation	Human sample preparation<br>Human frozen BM samples were thawed at 37°C and then transferred to 10 mL of thawing media (IMDM, 10% FCS, 1 mM EDTA, 1:1000 DNase). They were subsequently resuspended in StemSpan media (StemSpan™ SFEM II #09605) and allowed to recover in the incubator for 20 minutes. Following this, the cells were resuspended in 500 µL of antibody mix (Lin(CD3/14/16/19/20/56)-APC; CD38-Pe/Cy7, CD34-FITC) and incubated at 4°C for 45 minutes. After the incubation, the cells were washed with 0.9% NaCl and incubated with Zombie-Aqua (1:1000) for 10 minutes at room temperature. Finally, the cells were washed and resuspended in 0.9% NaCl, and filtered for FACS sorting.<br><br>Mouse sample preparation<br>C57BL/6J mice were euthanized by CO2 inhalation followed by cervical dislocation according to guidelines and animal protocols approved by the German authorities. Using forceps and scissors, legs and spines were dissected. To isolate femurs, tibiae, iliae, and vertebrae, connective tissue was removed with a scalpel. The isolated bones were gently crushed twice with 5 mL of ice-cold PBS (Sigma, # D8537) using a mortar and pestle and the cell suspension was then filtered through a 40 um sterile filter (Corning, #352340) into a 50 mL falcon tube. Cells were subsequently erylyzed with ACK Lysis Buffer (Lonza, #10-548E) and washed with ice-cold PBS. Lineage depletion was performed using the Dynabeads™ Untouched™ Mouse CD4 Cells Kit (Invitrogen, #11415D). Cells were then stained with the following antibodies: Lin(CD4, CD8a, Gr1, Ter119, B220, CD11b) - BV650; c-Kit - BV711, Sca-1 - APC/Cy7; CD150 - PeDazzle; CD48 - PeCy7; CD34 - FITC) for 45 minutes at 4°C. After staining, cells were washed with 0.9% NaCl and filtered for FACS sorting.
Instrument	For cell sorting: FACS Aria Fusion (Becton Dickinson)<br>For analysis: LSRII, LSR Fortessa (Becton Dickinson)
Software	Analysis was performed with FlowJo, statistical analysis with Graphpad Prism.
Cell population abundance	Population abundance for omics analysis ranged from 1,000 to 5,000 cells, and for functional analysis, from 1 to 5,000 cells, as reported in the figures.
Gating strategy	For human samples, hematopoietic stem and progenitor cells (HSPCs) were gated as lineage– CD34+ CD38–, and progenitors as lineage– CD34+ CD38+. For mouse samples, HSCs were defined as Lin– Sca1+ c-Kit+ (LSK) CD150+ CD48–, or as LSK CD150+ CD48– CD34– and progenitors as Lin– Sca1– c-Kit+ (LS–K).

☒ Tick this box to confirm that a figure exemplifying the gating strategy is provided in the Supplementary Information.
