# Supplementary material for: Differentiation, ageing and leukaemia alter the metabolic profile of human bone marrow haematopoietic stem and progenitor cells
Source: Nat Cell Biol. 2025 Jul 15;27(8):1367–80. doi: 10.1038/s41556-025-01709-7 (PMC12339397; doi:10.1038/s41556-025-01709-7)
Supplement: Supplementary file 11 — Lipidomics method reporting checklist. [file 41556_2025_1709_MOESM11_ESM.pdf]

Contents of Report

Created by https://lipidomicstandards.org, version v2.4.0

Separation Workflow 1
Overall study design 1
Lipid extraction 1
Analytical platform 1
Quality control 1
Method qualification and validation 2
Reporting 2

Sample Descriptions 2
human bone marrow fresh / Human / Tissues (e.g., liver, heart, brain) 2
human bone marrow frozen / Human / Tissues (e.g., liver, heart, brain) 2
human bone marrow SCT / Human / Tissues (e.g., liver, heart, brain) 3
murine bone marrow fresh / Mouse / Tissues (e.g., liver, heart, brain) 3

Lipid Class Descriptions 3
1) CAR[M]+ / Lipid identification 3
1) CAR[M]+ / Lipid quantification 3
2) PC[M+H]+ / Lipid identification 4
2) PC[M+H]+ / Lipid quantification 4
3) PE[M+H]+ / Lipid identification 4
3) PE[M+H]+ / Lipid quantification 5
4) PC O[M+H]+ / Lipid identification 5
4) PC O[M+H]+ / Lipid quantification 5
5) SM[M+H]+ / Lipid identification 6
5) SM[M+H]+ / Lipid quantification 6
6) PE O[M+H]+ / Lipid identification 6
6) PE O[M+H]+ / Lipid quantification 7
7) PI[M+Na]+ / Lipid identification 7
7) PI[M+Na]+ / Lipid quantification 7
8) PI[M+NH4]+ / Lipid identification 8
8) PI[M+NH4]+ / Lipid quantification 8
9) TG[M+NH4]+ / Lipid identification 8
9) TG[M+NH4]+ / Lipid quantification 9
10) FC[M-OH]+ / Lipid identification 9
10) FC[M-OH]+ / Lipid quantification 9
11) LPC[M+H]+ / Lipid identification 10
11) LPC[M+H]+ / Lipid quantification 10
12) PS[M+H]+ / Lipid identification 10
12) PS[M+H]+ / Lipid quantification 11
13) HexCer[M+H]+ / Lipid identification 11
13) HexCer[M+H]+ / Lipid quantification 11

Separation Workflow

Overall study design

Title of the study	Metabolic profile of human bone marrow hematopoietic stem and progenitor cells upon differentiation, aging and leukemia		
Document creation date	03/04/2025	Corresponding Email	buescher@ie-freiburg.mpg.de
Principal investigator	Nina Cabezas-Wallscheid	Is the workflow targeted or untargeted?	Untargeted
Institution	MPI of Immunobiology and Epigenetics	Clinical	No

Lipid extraction

Extraction method	1-phase system	Were internal standards added prior extraction?	No
pH adjustment	None	Special conditions	FACS sorting into solvent mix
1-phase system	2-propanol + acetonitrile + water	Derivatization	-

Analytical platform

Ionizaton additives	Ammonium formate	MS Level	MS2
Number of separation dimensions	One dimension	Mass window for precursor ion isolation (in Da total isolation window)	2
Separation type 1	LC	Mass resolution for detected ion at MS2	High resolution
Separation mode 1 (liquid)	RP	Resolution at m/z 200 at MS2	35000
Detector	Mass spectrometer	Mass accuracy in ppm at MS2	5
MS type	QTOF	Recording mode of raw data at MS2	Centroid mode
MS vendor	Bruker	Was/Were additional dimension/techniques used	No
Ion source	ESI		

Quality control

Blanks	Yes	Quality control	Yes
Type of Blanks	Extraction blank	Type of QC sample	Sample pool

Method qualification and validation

Method validation	No

Reporting

Are reported raw data uploaded into repository?	Yes	Summary data	-
Link to repository / ID to entry	MassIVE MSV000097228	Raw data upload	Yes
Are metadata available?	No	Additional comments	Data on MassIVE will be made public upon publication of manuscript

Sample Descriptions

human bone marrow fresh / Human / Tissues (e.g., liver, heart, brain)

Perfusion	No	Additives	EDTA
Storage and collection conditions	Available	Were samples stored under inert gas?	No
Provided preanalytical information	-	Additional preservation methods	No
Temperature handling original sample	Room temperature	Biobank samples	No
Instant sample preparation	Yes	Sample homogenization	No
Storage temperature	4-8 °C		

human bone marrow frozen / Human / Tissues (e.g., liver, heart, brain)

Perfusion	No	Storage time (month)	12
Storage and collection conditions	Available	Freeze-thaw cycles	0
Provided preanalytical information	Time to freeze (min), Storage time (month), Freeze-thaw cycles	Additives	EDTA
Temperature handling original sample	Room temperature	Were samples stored under inert gas?	No
Instant sample preparation	No	Additional preservation methods	Yes
Time to freeze (min)	45	Type of preservation method	10% DMSO in FCS
Snap freezing in liquid N2	No	Biobank samples	No
Storage temperature	Liquid nitrogen	Sample homogenization	No

human bone marrow SCT / Human / Tissues (e.g., liver, heart, brain)

Perfusion	No	Storage and collection conditions	Unknown

murine bone marrow fresh / Mouse / Tissues (e.g., liver, heart, brain)

Perfusion	No	Additives	None
Storage and collection conditions	Available	Were samples stored under inert gas?	No
Provided preanalytical information	-	Additional preservation methods	No
Temperature handling original sample	4-8 °C	Biobank samples	No
Instant sample preparation	Yes	Sample homogenization	No
Storage temperature	4-8 °C		

Lipid Class Descriptions

1) CAR[M]+ / Lipid identification

Lipid class	CAR	Limit of detection	No
MS Level for identification	MS2	RT verified by standard	Yes
Identification level	Species level	Separation of isobaric/isomeric interferece confirmed	No
Polarity mode	Positive	Model for separation prediction	No
Type of positive (precursor)ion	[M]+	Additional dimension/techniques	-
Fragments for identification		Lipid Identification Software	Metaboscape
Isotope correction at MS2	No	Data manipulation	Centroiding
MS2 verified by standard	No	Nomenclature for intact lipid molecule	No
Background check at MS2	No	Nomenclature for fragment ions	N/A
Did you presume assumptions for identification?	No	Further identification remarks	-
Check on:	-		

Fragment name
headgroup

1) CAR[M]+ / Lipid quantification

Quantitative	No	Batch correction	No
Normalization to reference	No	Further quantification remarks	-

2) PC[M+H]+ / Lipid identification

Lipid class	PC	Limit of detection	No
MS Level for identification	MS2	RT verified by standard	No
Identification level	Species level	Separation of isobaric/isomeric interferece confirmed	No
Polarity mode	Positive	Model for separation prediction	No
Type of positive (precursor)ion	[M+H]+	Additional dimension/techniques	-
Fragments for identification		Lipid Identification Software	Metaboscape
Isotope correction at MS2	No	Data manipulation	Centroiding
MS2 verified by standard	No	Nomenclature for intact lipid molecule	No
Background check at MS2	No	Nomenclature for fragment ions	N/A
Did you presume assumptions for identification?	No	Further identification remarks	-
Check on:	-		

Fragment name
HG(PC,184)

2) PC[M+H]+ / Lipid quantification

Quantitative	No	Batch correction	No
Normalization to reference	No	Further quantification remarks	-

3) PE[M+H]+ / Lipid identification

Lipid class	PE	Limit of detection	No
MS Level for identification	MS2	RT verified by standard	Yes
Identification level	Species level	Separation of isobaric/isomeric interferece confirmed	No
Polarity mode	Positive	Model for separation prediction	No
Type of positive (precursor)ion	[M+H]+	Additional dimension/techniques	-
Fragments for identification		Lipid Identification Software	Metaboscape

Fragment name
-HG(PE,141)

Isotope correction at MS2	No	Data manipulation	Centroiding
MS2 verified by standard	No	Nomenclature for intact lipid molecule	No
Background check at MS2	No	Nomenclature for fragment ions	N/A
Did you presume assumptions for identification?	No	Further identification remarks	-
Check on:	-		

3) PE[M+H]+ / Lipid quantification

Quantitative	No	Batch correction	No
Normalization to reference	No	Further quantification remarks	-

4) PC O[M+H]+ / Lipid identification

Lipid class	PC O	Limit of detection	No
MS Level for identification	MS2	RT verified by standard	No
Identification level	Species level	Separation of isobaric/isomeric interferece confirmed	No
Polarity mode	Positive	Model for separation prediction	No
Type of positive (precursor)ion	[M+H]+	Additional dimension/techniques	-
Fragments for identification		Lipid Identification Software	Metaboscape

Fragment name
HG(PC,184)

Isotope correction at MS2	No	Data manipulation	Centroiding
MS2 verified by standard	No	Nomenclature for intact lipid molecule	No
Background check at MS2	No	Nomenclature for fragment ions	N/A
Did you presume assumptions for identification?	No	Further identification remarks	-
Check on:	-		

4) PC O[M+H]+ / Lipid quantification

Quantitative	No	Batch correction	No
Normalization to reference	No	Further quantification remarks	-

5) SM[M+H]+ / Lipid identification

Lipid class	SM	Limit of detection	No
MS Level for identification	MS2	RT verified by standard	No
Identification level	Species level	Separation of isobaric/isomeric interferece confirmed	No
Polarity mode	Positive	Model for separation prediction	No
Type of positive (precursor)ion	[M+H]+	Additional dimension/techniques	-
Fragments for identification		Lipid Identification Software	Metaboscape

Fragment name
HG(PC,184)

Isotope correction at MS2	No	Data manipulation	Centroiding
MS2 verified by standard	No	Nomenclature for intact lipid molecule	No
Background check at MS2	No	Nomenclature for fragment ions	N/A
Did you presume assumptions for identification?	No	Further identification remarks	-
Check on:	-		

5) SM[M+H]+ / Lipid quantification

Quantitative	No	Batch correction	No
Normalization to reference	No	Further quantification remarks	-

6) PE O[M+H]+ / Lipid identification

Lipid class	PE O	Limit of detection	No
MS Level for identification	MS2	RT verified by standard	No
Identification level	Species level	Separation of isobaric/isomeric interferece confirmed	No
Polarity mode	Positive	Model for separation prediction	No
Type of positive (precursor)ion	[M+H]+	Additional dimension/techniques	-
Fragments for identification	Fragment name: -HG(PE,141)	Lipid Identification Software	Metaboscape
Isotope correction at MS2	No	Data manipulation	Centroiding
MS2 verified by standard	No	Nomenclature for intact lipid molecule	No
Background check at MS2	No	Nomenclature for fragment ions	N/A
Did you presume assumptions for identification?	No	Further identification remarks	-
Check on:	-		

6) PE O[M+H]+ / Lipid quantification

Quantitative	No	Batch correction	No
Normalization to reference	No	Further quantification remarks	-

7) PI[M+Na]+ / Lipid identification

Lipid class	PI	Limit of detection	No
MS Level for identification	MS2	RT verified by standard	Yes
Identification level	Species level	Separation of isobaric/isomeric interferece confirmed	No
Polarity mode	Positive	Model for separation prediction	No
Type of positive (precursor)ion	[M+Na]+	Additional dimension/techniques	-
Fragments for identification	Fragment name: -HG(PI,260+Na)	Lipid Identification Software	Metaboscape
Isotope correction at MS2	No	Data manipulation	Centroiding
MS2 verified by standard	No	Nomenclature for intact lipid molecule	No
Background check at MS2	No	Nomenclature for fragment ions	N/A
Did you presume assumptions for identification?	No	Further identification remarks	-
Check on:	-		

7) PI[M+Na]+ / Lipid quantification

Quantitative	No	Batch correction	No
Normalization to reference	No	Further quantification remarks	-

8) PI[M+NH4]+ / Lipid identification

Lipid class	PI	Limit of detection	No
MS Level for identification	MS2	RT verified by standard	No
Identification level	Species level	Separation of isobaric/isomeric interferece confirmed	No
Polarity mode	Positive	Model for separation prediction	No
Type of positive (precursor)ion	[M+NH4]+	Additional dimension/techniques	-
Fragments for identification	Fragment name: -HG(PI,260+NH4)	Lipid Identification Software	Metaboscape
Isotope correction at MS2	No	Data manipulation	Centroiding
MS2 verified by standard	No	Nomenclature for intact lipid molecule	No
Background check at MS2	No	Nomenclature for fragment ions	N/A
Did you presume assumptions for identification?	No	Further identification remarks	-
Check on:	-		

8) PI[M+NH4]+ / Lipid quantification

Quantitative	No	Batch correction	No
Normalization to reference	No	Further quantification remarks	-

9) TG[M+NH4]+ / Lipid identification

Lipid class	TG	Limit of detection	No
MS Level for identification	MS2	RT verified by standard	No
Identification level	Species level	Separation of isobaric/isomeric interferece confirmed	No
Polarity mode	Positive	Model for separation prediction	No
Type of positive (precursor)ion	[M+NH4]+	Additional dimension/techniques	-
Fragments for identification		Lipid Identification Software	Metaboscape

Fragment name
-FA1(+HO)-(NH3)
-FA2(+HO)-(NH3)
-FA3(+HO)-(NH3)

Isotope correction at MS2	No	Data manipulation	Centroiding
MS2 verified by standard	No	Nomenclature for intact lipid molecule	No
Background check at MS2	No	Nomenclature for fragment ions	N/A
Did you presume assumptions for identification?	No	Further identification remarks	-
Check on:	-		

9) TG[M+NH4]+ / Lipid quantification

Quantitative	No	Batch correction	No
Normalization to reference	No	Further quantification remarks	-

10) FC[M-OH]+ / Lipid identification

Lipid class	FC	Limit of detection	No
MS Level for identification	MS2	RT verified by standard	Yes
Identification level	Species level	Separation of isobaric/isomeric interferece confirmed	No
Polarity mode	Positive	Model for separation prediction	No
Type of positive (precursor)ion	[M-OH]+	Additional dimension/techniques	-
Fragments for identification		Lipid Identification Software	Metaboscape

Fragment name
C6H9
C10H15
C12H17
C13H19

Isotope correction at MS2	No	Data manipulation	Centroiding
MS2 verified by standard	No	Nomenclature for intact lipid molecule	No
Background check at MS2	No	Nomenclature for fragment ions	N/A
Did you presume assumptions for identification?	No	Further identification remarks	-
Check on:	-		

10) FC[M-OH]+ / Lipid quantification

Quantitative	No	Batch correction	No
Normalization to reference	No	Further quantification remarks	-

11) LPC[M+H]+ / Lipid identification

Lipid class	LPC	Limit of detection	No
MS Level for identification	MS2	RT verified by standard	No
Identification level	Species level	Separation of isobaric/isomeric interferece confirmed	No
Polarity mode	Positive	Model for separation prediction	No
Type of positive (precursor)ion	[M+H]+	Additional dimension/techniques	-
Fragments for identification		Lipid Identification Software	Metaboscape

Fragment name
HG(PC,184)

Isotope correction at MS2	No	Data manipulation	Centroiding
MS2 verified by standard	No	Nomenclature for intact lipid molecule	No
Background check at MS2	No	Nomenclature for fragment ions	N/A
Did you presume assumptions for identification?	No	Further identification remarks	-
Check on:	-		

11) LPC[M+H]+ / Lipid quantification

Quantitative	No	Batch correction	No
Normalization to reference	No	Further quantification remarks	-

12) PS[M+H]+ / Lipid identification

Lipid class	PS	Limit of detection	No
MS Level for identification	MS2	RT verified by standard	No
Identification level	Species level	Separation of isobaric/isomeric interferece confirmed	No
Polarity mode	Positive	Model for separation prediction	No
Type of positive (precursor)ion	[M+H]+	Additional dimension/techniques	-
Fragments for identification		Lipid Identification Software	Metaboscape

Fragment name
-HG(PS,185)

Isotope correction at MS2	No	Data manipulation	Centroiding
MS2 verified by standard	No	Nomenclature for intact lipid molecule	No
Background check at MS2	No	Nomenclature for fragment ions	N/A
Did you presume assumptions for identification?	No	Further identification remarks	-
Check on:	-		

12) PS[M+H]+ / Lipid quantification

Quantitative	No	Batch correction	No
Normalization to reference	No	Further quantification remarks	-

13) HexCer[M+H]+ / Lipid identification

Lipid class	HexCer	Limit of detection	No
MS Level for identification	MS2	RT verified by standard	No
Identification level	Species level	Separation of isobaric/isomeric interferece confirmed	No
Polarity mode	Positive	Model for separation prediction	No
Type of positive (precursor)ion	[M+H]+	Additional dimension/techniques	-
Fragments for identification		Lipid Identification Software	Metaboscape

Fragment name
-HG(Hex,180)

Isotope correction at MS2	No	Data manipulation	Centroiding
MS2 verified by standard	No	Nomenclature for intact lipid molecule	No
Background check at MS2	No	Nomenclature for fragment ions	N/A
Did you presume assumptions for identification?	No	Further identification remarks	-
Check on:	-		

13) HexCer[M+H]+ / Lipid quantification

Quantitative	No	Batch correction	No
Normalization to reference	No	Further quantification remarks	-
